# Plant-Derived Proteins and Peptides as Potential Immunomodulators

**DOI:** 10.3390/molecules29010209

**Published:** 2023-12-29

**Authors:** Iwona Szerszunowicz, Szymon Kozicki

**Affiliations:** Department of Food Biochemistry, University of Warmia and Mazury in Olsztyn, Plac Cieszyński 1, 10-726 Olsztyn-Kortowo, Poland

**Keywords:** BIOPEP-UWM database, immunomodulators/immunopeptides, in silico proteolysis, ProtParam tool

## Abstract

The immune response of humans may be modulated by certain biopeptides. The present study aimed to determine the immunomodulatory potential of plant-derived food proteins and hydrolysates obtained from these proteins via monocatalytic in silico hydrolysis (using ficin, stem bromelainm or pepsin (pH > 2)). The scope of this study included determinations of the profiles of select bioactivities of proteins before and after hydrolysis and computations of the frequency of occurrence of selected bioactive fragments in proteins (parameter A), frequency/relative frequency of the release of biopeptides (parameters A_E_, W) and the theoretical degree of hydrolysis (DH_t_), by means of the resources and programs available in the BIOPEP-UWM database. The immunomodulating (ImmD)/immunostimulating (ImmS) peptides deposited in the database were characterized as well (ProtParam tool). Among the analyzed proteins of cereals and legumes, the best precursors of ImmD immunopeptides (YG, YGG, GLF, TPRK) turned out to be rice and garden pea proteins, whereas the best precursors of ImmS peptides appeared to be buckwheat (GVM, GFL, EAE) and broad bean (LLY, EAE) proteins. The highest number of YG sequences was released by stem bromelain upon the simulated hydrolysis of rice proteins (AE = 0.0010–0.0820, W = 0.1994–1.0000, DH_t_ = 45–82%). However, antibacterial peptides (IAK) were released by ficin only from rice, oat, and garden pea proteins (DH_t_ = 41–46%). Biopeptides (YG, IAK) identified in protein hydrolysates are potential immunomodulators, nutraceuticals, and components of functional food that may modulate the activity of the human immune system. Stem bromelain and ficin are also active components that are primed to release peptide immunomodulators from plant-derived food proteins.

## 1. Introduction

The human immune system plays a key role in the identification and removal of not only various potentially adverse substances (pathogens) but also aging and cancer cells [1,2,3]. It also mediates the control of inflammatory conditions [4,5], whereas its dysfunctions initiate the development of autoimmune diseases [3,6], including multiple sclerosis (MS) [3,7], rheumatoid arthritis (RS), systemic lupus erythematosus (SLE) [3,8], and type 1 diabetes mellitus (T1D) [3,9,10].

The immune system functions based on two major mechanisms: innate (natural/native) and adaptive (specific/acquired) immunity [1,2], while changes proceeding in one of these two immunity types or in both are referred to as immunomodulation [6]. These two sub-subsystems are crucial not only for the immunomodulation process but also for compounds, including components of plant materials and foods that are likely to affect the immune response [6].

Innate immunity is the first non-specific sub-system (the first line of defense against pathogens) capable of stimulating and developing long-term antigen-specific immunity [2]. Its main constituents include epithelial barriers (skin, mucosal membranes), effector cells (monocytes/macrophages, natural killer (NK) cells, dendritic cells (DCs), mast cells, neutrophils, innate lymphoid cells, eosinophils), and pattern recognition receptors (PRRs) [1,2], while its minor elements are represented by inflammatory components like cytokines, including, e.g., tumor necrosis factor (TNF)—one of the proinflammatory and cytotoxic cytokines, interleukin-1 (IL-1), IL-12, interferon gamma (IFN-gamma), and defensins [1,2].

The second mechanism of the immune system is adaptive (specific) immunity, namely highly specific humoral (mediated by B lymphocytes) or cellular (mediated by T lymphocytes) immunity [1,2]. The B cells recognize an antigen via a B-cell receptor (BCR) or as a soluble component, i.e., secretory BCRs called “antibodies”, which may directly bind blood serum antigens [2]. In turn, the cellular response involves binding T lymphocytes with a pathogen or infected cells, followed by the lysis of these cells and release of immunity-regulating cytokines [1]. The T cells recognize (via the T-cell receptor (TCR)) the peptide fragments (epitopes) present on the cell surface as cell surface receptors (major histocompatibility complex (MHC) class I/class II (MHCI/MHCII)) [2]. Due to their functions and expression of surface proteins, the T cells are divided into, e.g., cytotoxic T cells expressing a surface receptor, or clusters of differentiation (CD)8+, which—once activated—may proliferate and kill infected or neoplastic cells [1]. They also include helper T (Th) and regulatory T (suppressor T, Ts) cells. The first ones exhibit the expression of a surface receptor CD4+ and recognize exogenous antigens complexed with MHCII. They also secrete cytokines and enable the activation of immune system cells, including B and T lymphocytes. In turn, the Ts cells prevent the adverse activation of the immune system or the development of autoimmune diseases [1].

Immunomodulation is indispensable for controlling immune system activity because its dysregulation results in the development of autoimmune diseases, whereas an inappropriate response to non-harmful antigens leads to the development of allergies or hypersensitivity [3]. Given the complexity of the immune system alone as well as biochemical process/pathways occurring therein, the term “immunomodulation” refers to a broad spectrum of changes in the innate or/and acquired immunity [6]. In turn, the agents/compounds/drugs affecting the immune system are referred to as immunomodulators, which—depending on their activities in this system—are classified as immunosuppressants (inhibition or blocking), immunostimulants (stimulation or activation) [1], and immunoadjuvants (substances enhancing immunogenicity of vaccine preparations) [1,11]. The compounds that inhibit or block the immune system are applied in the case of, e.g., autoimmune diseases, allergy/asthma, inflammation, or transplantation. In turn, immunostimulants (immunoactivators) activate the immune system and are deployed to, e.g., eliminate viral infections or neoplasms [3]. Drugs modulating the immune system activity include, i.a., cyclosporine A, levamisole [1,12], imiquimod, pidotimod, tilorone, cyclophosphamide, prostaglandin [1], aristolochic acid [1,12], and tacrolimus [12]. The immune response may also be modulated by compounds such as, e.g., vitamins (C, D_3_, E, B_12_), minerals (selenium, zinc), hydrolyzed rice bran (rice bran arabinoxylan complex or biobran), compounds synthesized by plants (phytonutrients) like, e.g., Aloe vera polysaccharides, curcumin, isoflavones, isoflavones (i.e., primarily daidzein and genistein), and phenolic compounds (including resveratrol), whose bioactivity has been confirmed in clinical trials on patients administered their appropriate daily oral doses [13].

Another group of compounds capable of modulating the activity of the immune system are also peptides [3] (immunopeptides) [6] and peptidomimetics (compounds, in which the pharmacophore mimics a peptide or a protein in a three-dimensional space to interact with a biological target and trigger a biological effect), that enable the development of immunological tolerance [3]. Immunopeptides represent one of the most diversified and complex groups of biologically active peptides (BAPs, biopeptides (BPs)) and often share multiple functions (multi-functional peptides). Considering the process they affect, their immunomodulating effects may be classified as proliferative/antiproliferative, proinflammatory/anti-inflammatory, and cytoprotective/cytotoxic. Proliferative/antiproliferative peptides participate in the stimulation (proliferative) or prevention (antiproliferative) of cancer metastases and spread (sometimes they are called carcinogenic or anticarcinogenic peptides). In turn, proinflammatory/anti-inflammatory peptides enhance or prevent/alleviate inflammatory processes, whereas the cytoprotective/cytotoxic peptides take part in apoptosis regulation (they are also referred to as apoptotic or antiapoptotic peptides) [6].

Peptide immunomodulators (immunopeptides), including those of plant origin, can module the human immune response by activating macrophages, stimulating phagocytosis [12,14,15,16,17,18], increasing the number of leukocytes and immunomodulators (e.g., cytokines, nitric oxide (NO), immunoglobulins), or enhancing the stimulation of splenocytes, CD4+, CD8+, CD11b+, and CD56+ cells [12,14,15]. Immunopeptides are classified as BPs [6,16]. Biopeptides usually contain from 2 to 20 amino acid residues [12,14,15,16,17,18,19,20,21,22,23], but some works have reported even higher residue numbers [1,6,14,15,17,18,20,21]. The BPs show a broad spectrum of bioactivities and include not only peptides featuring immunomodulatory activity [1,6,12,14,15,16,17,18] but also these exhibiting antihypertensive [14,17,18,20,21,22], antioxidant [1,14,17,18,20,21,23], anticarcinogenic [1,14,17,18,21,22], antidiabetic [20,21,22], antimicrobial [1,14,17,21], anti-inflammatory [15,17,18], hypocholesterolemic [14,17,18,21,22,24], opioid-like [14], and anti-aging [12] activities, while some of them exhibit multi-faceted activity [1,15,18,20,21].

The BPs are fragments of amino acid sequences (inactive) present in proteins (including food-derived proteins), from which they may be released and then exhibit a certain bioactivity [12,18,19,20,21,22,25]. They may be released from food-derived proteins during various technological processes applied for food production and processing [12,17,18,19,22] and for microbiological fermentation [12,14,17,18,19,20,21,22]. BPs may also be synthesized under physiological conditions of the human gastrointestinal tract upon the activity of endogenous proteolytic enzymes [18,19,20,22]. Furthermore, biopeptides are constituents of functional foods [15,18,19], infant formulas [12,15,19], clinical nutrition products [15], sport nutrition products [15], and dietary supplements [19]. They are also components of protein hydrolysates produced using specific proteolytic enzymes that enable synthesizing biocompounds with a desired bioactivity profile [14,17,18,19,20,21]. Such hydrolysates may then be used to isolate and purify peptides with a specified bioactivity [12,14,17,18,23,24,25]. This bioactivity (involving interactions with body receptors in order to induce a specified biological effect) [18] correlates primarily with the chemical structure of these compounds [14,16,18,19,20,21,23,24,26].

The present study addressed plant-derived proteins from crops due to the hypothesis assuming that, most likely, crops have lesser autoimmune potential (a lower value of the Gershteyn–Ferreira (GF) index) [6,27], and that their proteins may serve as natural sources of bioactive peptides, including those affecting immune response modulation [12,14,15,16,17,18]. The bioactivity of proteins and peptides is evaluated by means of, i.e., bioinformatic tools, like—especially recently—the BIOPEP-UWM database [28], which provide not only amino acid sequences of food proteins and bioactive peptides but also appropriate bioinformatic tools, enabling, e.g., protein evaluation in terms of its bioactivity, proteolysis simulations exploiting the specificity of action of proteolytic enzymes deposited in this database, and assessment of the bioactivity of the products of in silico hydrolysis [28].

The present study aimed to determine the immunomodulatory potential of selected plant-derived food proteins and hydrolysates obtained from these proteins via monocatalytic simulations of the release of peptide bonds using ficin, stem bromelain or pepsin (pH > 2).

## 2. Results

**Figure 1:**
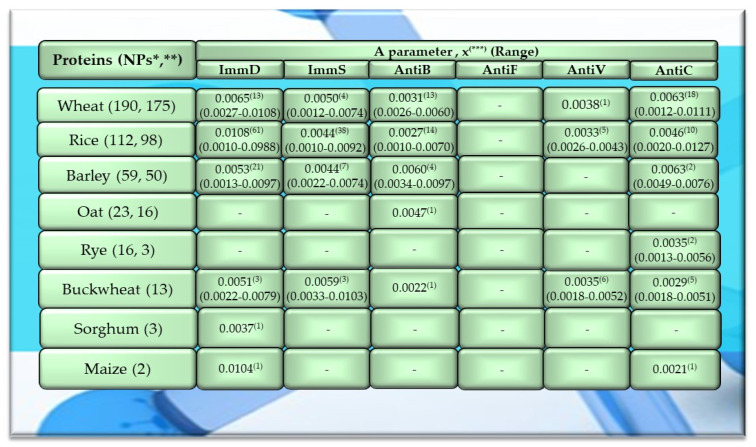
Characteristics of selected activities of cereal proteins. ImmD—immunomodulatory, ImmS—immunostimulatory, AntiB—antibacterial, AntiF—antifungal, AntiV—antiviral, AntiC—anticarcinogenic activity. * NPs—The number of total proteins including all protein fragments available from the BIOPEP-UWM database, **—the number of proteins or protein fragments meeting the molecular criterion, i.e., possessing at least 100 amino acid residues, comprising the A parameter (the frequency of bioactive fragment occurrence in a protein sequence, defined in the “Materials and Methods” section), x^(^***^)^—mean value (*** numbers in superscripts indicate the number of proteins in which fragments of bioactive sequences were identified using the BIOPEP-UWM database programs). This figure was prepared based on the data/results provided in Table A1, Table A2, Table A3, Table A4 and Table A5 (attached in Appendix A).

**Figure 2:**
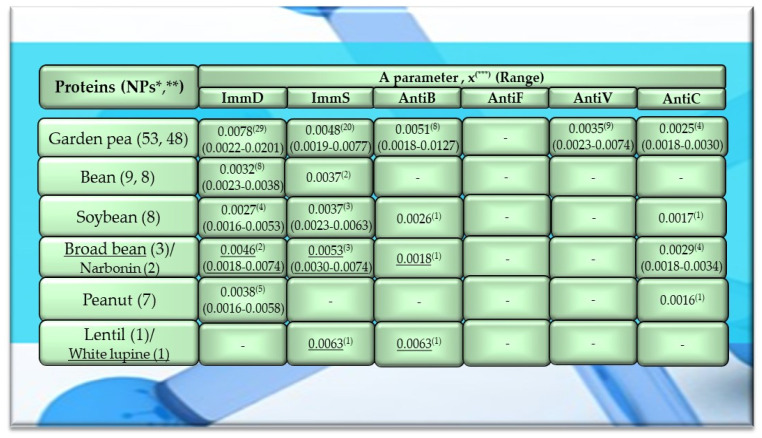
Characteristics of the selected activities of legume proteins. ImmD—immunomodulatory, ImmS—immunostimulatory, AntiB—antibacterial, AntiF—antifungal, AntiV—antiviral, AntiC—anticarcinogenic activity. * NPs—The number of total proteins including all protein fragments available from the BIOPEP-UWM database, **—the number of proteins or protein fragments meeting the molecular criterion, i.e., possessing at least 100 amino acid residues, comprising the A parameter (the frequency of bioactive fragment occurrence in a protein sequence, defined in the “Materials and Methods” section), x^(^***^)^—mean value (*** numbers in superscripts indicate the number of proteins in which fragments of bioactive sequences were identified using the BIOPEP-UWM database programs). This figure was prepared based on the data/results provided in Table A6, Table A7, Table A8, Table A9 and Table A10 (attached in Appendix A).

**Figure 3:**
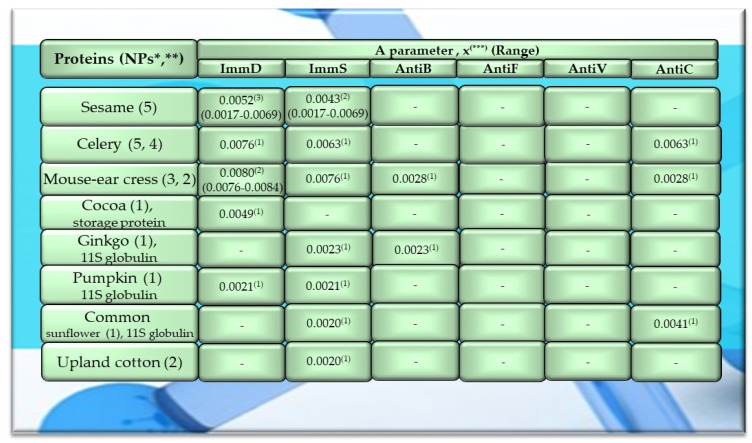
Characteristics of selected activities of other plant proteins. ImmD—immunomodulatory, ImmS—immunostimulatory, AntiB—antibacterial, AntiF—antifungal, AntiV—antiviral, AntiC—anticarcinogenic activity. * NPs—The number of total proteins including all protein fragments available from the BIOPEP-UWM database, **—the number of proteins or protein fragments meeting the molecular criterion, i.e., possessing at least 100 amino acid residues, the A parameter (the frequency of bioactive fragment occurrence in a protein sequence, defined in the “Materials and Methods” section), x^(^***^)^—mean value (*** numbers in superscripts indicate the number of proteins in which fragments of bioactive sequences were identified using the BIOPEP-UWM database programs). This figure was prepared based on the data/results provided in Table A11, Table A12 and Table A13 (attached in Appendix A).

**Figure 4:**
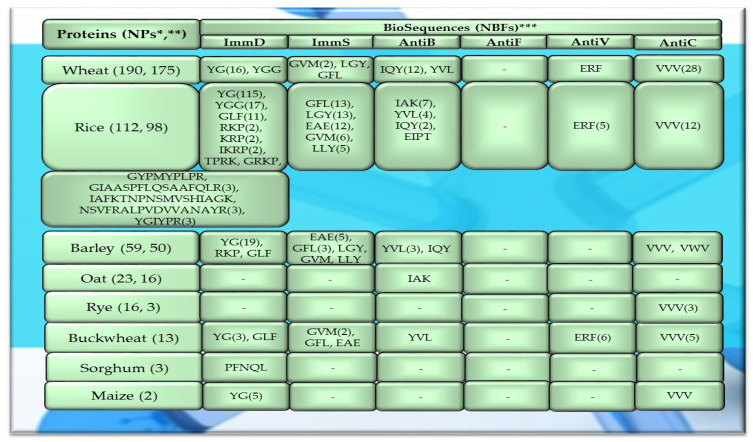
Fragments of amino acid protein sequences with potential activities modulating the immune system found in cereal proteins. ImmD—immunomodulatory, ImmS—immunostimulatory, AntiB—antibacterial, AntiF—antifungal, AntiV—antiviral, AntiC—anticarcinogenic activity. * NPs—The number of total proteins including all protein fragments available from the BIOPEP-UWM database, **—the number of proteins or protein fragments meeting the molecular criterion, i.e., possessing at least 100 amino acid residues, *** NBFs—the number of biofragments or biosequences presented in the form of a single-letter amino acid code: A—alanine, R—arginine, N—asparagine, D—aspartic acid, Q—glutamine, E—glutamic acid, G—glycine, H—histidine, I—isoleucine, L—leucine, K—lysine, M—methionine, F—phenylalanine, P—proline, S—serine, T—threonine, W—tryptophan, Y—tyrosine, V—valine. This figure was prepared based on the data/results provided in Table A1, Table A2, Table A3, Table A4 and Table A5 (attached in Appendix A).

**Figure 5:**
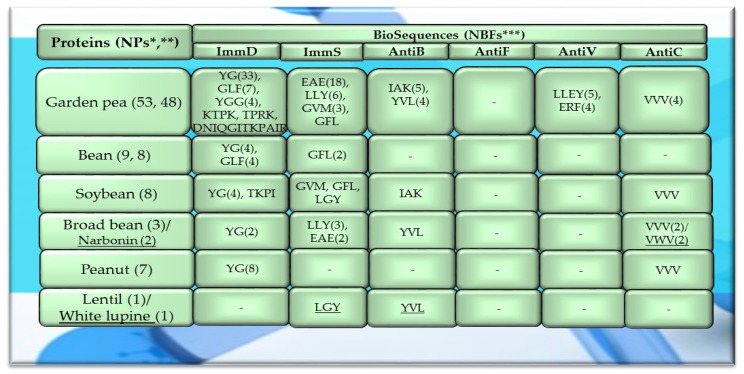
Fragments of amino acid protein sequences with potential activities modulating the immune system found in legume proteins. ImmD—immunomodulatory, ImmS—immunostimulatory, AntiB—antibacterial, AntiF—antifungal, AntiV—antiviral, AntiC—anticarcinogenic activity. * NPs—The number of total proteins including all protein fragments available from the BIOPEP-UWM database, **—the number of proteins or protein fragments meeting the molecular criterion, i.e., possessing at least 100 amino acid residues, *** NBFs—the number of biofragments or biosequences presented in the form of a single-letter amino acid code: A—alanine, R—arginine, N—asparagine, D—aspartic acid, Q—glutamine, E—glutamic acid, G—glycine, I—isoleucine, L—leucine, K—lysine, M—methionine, F—phenylalanine, P—proline, T—threonine, W—tryptophan, Y—tyrosine, V—valine. This figure was prepared based on the data/results provided in Table A6, Table A7, Table A8, Table A9 and Table A10 (attached in Appendix A).

**Figure 6:**
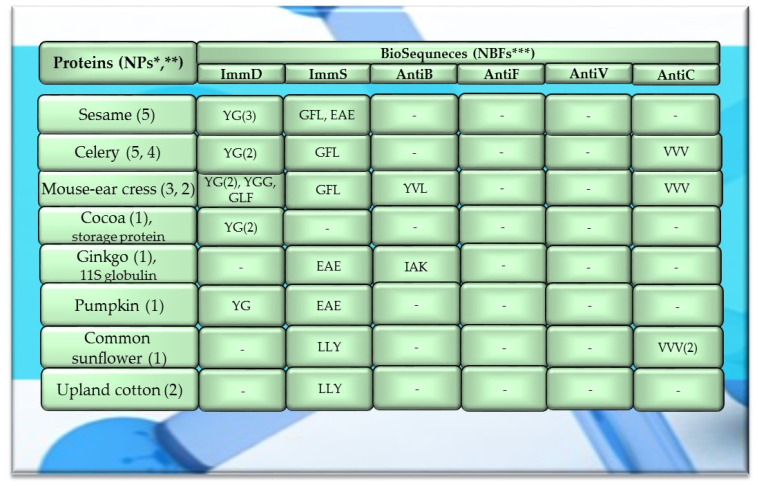
Fragments of amino acid protein sequences with potential activities modulating the immune system found in other plant proteins. ImmD—immunomodulatory, ImmS—immunostimulatory, AntiB—antibacterial, AntiF—antifungal, AntiV—antiviral, AntiC—anticarcinogenic activity. * NPs—The number of total proteins including all protein fragments available from the BIOPEP-UWM database, **—the number of proteins or protein fragments meeting the molecular criterion, i.e., possessing at least 100 amino acid residues, *** NBFs—the number of biofragments or biosequences presented in the form of a single-letter amino acid code: A—alanine, E—glutamic acid, G—glycine, I—isoleucine, L—leucine, K—lysine, F—phenylalanine, Y—tyrosine, V—valine. This figure was prepared based on the data/results provided in Table A11, Table A12 and Table A13 (attached in Appendix A).

Of the 756 proteins/protein fragments available in the BIOPEP-UWM database (https://biochemia.uwm.edu.pl/biopep-uwm) (accessed on 1 October 2023), 524 compounds of plant origin were selected for analysis. Taking into account that a protein is an amino acid polymer expected to contain at least 100 amino acid residues in its structure, such a molecular criterion was adopted while analyzing the selected proteins, especially including the protein sequence fragments. The plant proteins were grouped into those derived from cereals (wheat, rice, barley, oat, rye, buckwheat, sorghum, maize), legumes (garden pea, bean, soybean, broad bean/narbonin, peanut, lentil, white lupine), and others (sesame, celery, mouse-ear cress, cocoa, ginkgo, pumpkin, common sunflower, upland cotton, rape, serendipity berry). All the data and necessary information related to the analyzed compounds are provided in Table A1, Table A2, Table A3, Table A4, Table A5, Table A6, Table A7, Table A8, Table A9, Table A10, Table A11, Table A12 and Table A13, whereas Figure 1, Figure 2 and Figure 3 present the mean numeric values and the range of parameter A values (the frequency of occurrence of fragments with the selected bioactivities) computed for the grouped proteins and the number of proteins containing fragments of sequences exhibiting the analyzed bioactivities. The sequences of such bioactive fragments (found in the analyzed proteins) are presented in Figure 4, Figure 5 and Figure 6, which, like the other figures (Figure 1, Figure 2 and Figure 3), were prepared based on the results of bioinformatic analyses (ID numbers of the analyzed proteins, numeric values of parameter A, amino acid sequences of bioactive fragments) collated in Table A1, Table A2, Table A3, Table A4, Table A5, Table A6, Table A7, Table A8, Table A9, Table A10, Table A11, Table A12 and Table A13. A description and the scope of the conducted bioinformatic analyses are provided in the “Materials and Methods” section.

A protein or a protein fragment (meeting the molecular criterion, i.e., possessing at least 100 amino acid residues) which contained at least one fragment of the analyzed bioactive sequence (meeting experimental assumptions, i.e., exhibiting ImmD, ImmS, AntiB, AntiF, AntiV, or AntiC activity) was referred to as a precursor of biopeptides with a potential activity modulating the human immune system.

The values provided in the figures (Figure 1, Figure 2 and Figure 3) in the first columns and in brackets, e.g., wheat (190, 175), are denoted as follows: 190—the number of all protein sequences and protein fragments classified to the same cereal species, wheat, which were available in the BIOPEP-UWM database; 175—the number of proteins and protein fragments meeting the adopted molecular criterion (at least 100 amino acid residues in a molecule of the analyzed protein/fragment to enable its complete bioinformatic analysis; description in the “Materials and Methods” section). The IDs of proteins/protein fragments (including the numbers of amino acid residues in their molecules) that met and did not meet the adopted molecular criterion are provided in Table A1, Table A2, Table A3, Table A4, Table A5, Table A6, Table A7, Table A8, Table A9, Table A10, Table A11, Table A12 and Table A13.

The values provided in superscript and presented in the figures (Figure 1, Figure 2 and Figure 3) denote the number of analyzed compounds which contained fragments of amino acid sequences exhibiting the analyzed activities which were found using the BIOPEP-UWM database programs and for which the frequency of the occurrence of fragments of the amino acid sequence with a selected activity was computed in the analyzed protein (parameter A). Each figure footnote (Figure 1, Figure 2, Figure 3, Figure 4, Figure 5 and Figure 6) provides information demonstrating that the figure has been prepared based on the results of the analyses provided in a respective table (provided in “Appendix A” section).

However, not all of the analyzed proteins/protein fragments contained at least one fragment of the amino acid sequence with ImmD or ImmS or AntiB or AntiF or AntiV or AntiC activity (commonly defined as the activity affecting the immune system). If a protein/protein fragment possessed at least one fragment of the amino acid sequence with a given activity, it could be deemed a potential source of biopeptide/biopeptides with the searched bioactivity, but only fragments of sequences released from a protein sequence or a protein fragment could be defined as biopeptide/biopeptides (if they had been released upon, e.g., the action of proteolytic enzyme/enzymes). To enable this release, monocatalytic hydrolysis of each protein or its fragment was conducted using the specificity of action of ficin or stem bromelain or pepsin (pH > 2) (the description and scope of these analyses is provided in the “Materials and Methods” section), and the respective results are presented in the following manuscript section.

### 2.1. Characterization of Plant-Derived Proteins as Precursors of Peptides with Potential Immune System Modulating Activities (ImmD, ImmS, AntiB, AntiF, AntiV, AntiC)

Among the analyzed plant-derived proteins, the highest number of sequences was analyzed for wheat and rice proteins, accounting for 45.5% and 26.8% of all cereal proteins as well as 36.3% and 21.4% of all plant proteins available in the BIOPEP-UWM database resources, respectively (Figure 1). The cereal proteins had the highest number of biofragments with the ImmD activity. The mean parameter A value computed for rice proteins was A = 0.0108 (range 0.0010–0.0988), and this activity type was identified in 61 proteins of this protein group, which accounted for 54.5% of all rice proteins available in the BIOPEP-UWM database and for 62.2% the analyzed protein sequences of rice (Figure 1, Table A2). The highest numeric values of parameter A were found for rice prolamins (clone PPROL 7; 14; 4A) (ID(s) 1152, 1154, 1155), and also for other proteins of this cereal (glycine-rich protein (ID 1562), glycine-rich cell wall structural protein 2 (ID 1542)) (Table A2). The frequency of the occurrence of fragments of sequences with ImmD activity for maize legumin A (parameter A) reached 0.0104, whereas the mean numeric value of parameter A computed for ImmD activity and wheat proteins was A = 0.0065. However, only 7.4% of the analyzed proteins possessed fragments of an amino acid sequence exhibiting this activity. The barley and buckwheat proteins also contained fragments with ImmD activity (mean A values: A = 0.0053, A = 0.0051, respectively), which was identified in 42% and 23% of these proteins, respectively (Figure 1). Only the sequence of one oat protein (avenin precursor) and two rye proteins (omega-secalin and glutenin-HMW subunit) contained one and three fragments of sequences with AntiB and AntiC activity, respectively (Figure 1 and Figure 4, Table A4 and Table A5).

In turn, the mean value of parameter A computed for cereal proteins and ImmS activity was the highest in the case of buckwheat proteins (A = 0.0059), whereas in the case of barley proteins, the highest values of this parameter were calculated for AntiB activity (A = 0.0060). Wheat and barley proteins turned out to be better precursors of peptides with AntiC activity (A = 0.0063), but bioactive fragments were identified in 10.3% and 4% of these proteins, respectively (Figure 1, Table A1, Table A2, Table A3, Table A4 and Table A5).

Among the analyzed proteins of legumes, sequence fragments with ImmD and AntiV activities occurred (usually due to the high values of A parameter) only in garden pea proteins (mean A values: A = 0.0078, A = 0.0035, respectively), whereas ImmS and AntiC activities prevailed in broad bean legumins (mean A values: A = 0.0053, A = 0.0024, respectively), and AntiC activity (mean A values: 0.0034) only in narbonins (from Vicia narbonensis and Vicia pannonica) (Figure 2, Table A6 and Table A9). In turn, fragments of sequences with the ImmS activity were also (usually, A parameter range: 0.0053–0.0076) identified in proteins of mouse-ear cress, white lupine, celery, and broad beans (Figure 2 and Figure 3, Table A6, Table A7, Table A8, Table A9, Table A10, Table A11, Table A12 and Table A13).

Only wheat, rice, buckwheat, and garden pea proteins contained fragments of amino acid sequences exhibiting ImmD, ImmS, AntiB, AntiV, and AntiC activities. Fragments of amino acid sequences featuring the AntiF activity were not identified in any of the plant proteins, regardless of the plant genera and species (Figure 1, Figure 2 and Figure 3, Table A1, Table A2, Table A3, Table A4, Table A5, Table A6, Table A7, Table A8, Table A9, Table A10, Table A11, Table A12 and Table A13).

The sequences of the analyzed cereal proteins (wheat, rice, barley, buckwheat, maize), legume proteins (garden pea, bean, soybean, broad bean, peanut), and other proteins (sesame, celery, mouse-ear cress, cocoa, pumpkin) contained motifs of the YG sequence (written in the form of a single-letter amino acid code: tyrosine (Y) and glycine (G)) exhibiting ImmD activity. The highest number of YG fragments was identified in the proteins of rice—115, barley—19, wheat—16 (Figure 4), garden pea—33, and peanut—8 (Figure 5 and Figure 6). In turn, YGG fragments occurred in wheat, rice, garden pea, and mouse-ear cress proteins, with their highest number identified in rice and garden pea proteins, i.e., 17 and 4, respectively (Figure 4 and Figure 5). Rice proteins also contained fragments of sequences consisting of 6 and 16 amino acid residues that were identified in prolamins, fragments with 17 amino acid residues identified in glutelins, and a GYPMYPLPR (oryzatensin) fragment found in an allergenic protein (japonica cultivar group, ID 1576, Figure 4, Table A2). GLF fragments with ImmD activity were identified in rice, barley, buckwheat, garden peas, beans, and mouse-ear cress proteins. In turn, PFNQL occurred only in sorghum proteins and GRKP represented a fragment of a sequence of one rice protein, whereas TPRK was found in rice and garden pea proteins, and TKPI was found in soybean protein (Figure 4, Figure 5 and Figure 6, Table A1, Table A2, Table A3, Table A4, Table A5, Table A6, Table A7, Table A8, Table A9, Table A10, Table A11, Table A12 and Table A13).

The immunostimulating fragments GFL, EAE, and GVM occurred in proteins of rice, barley, buckwheat, and garden peas (Figure 4 and Figure 5, Table A2, Table A3, Table A4, Table A5 and Table A6). ImmS fragments sharing the same amino acid sequences and additionally containing other sequences, like LGY and LLY, also occurred in rice and barley proteins (Figure 4 and Figure 5, Table A2, Table A3, Table A4, Table A5 and Table A6). In turn, LGY fragments occurred in wheat, soybean, and white lupine proteins, whereas GVM was also found in wheat and soybean proteins, and LLY and EAE were found in broad bean proteins (Figure 4 and Figure 5, Table A1, Table A8 and Table A9). The antibacterial YVL sequences were not identified in oat and soybean proteins, whereas IQY was detected in the primary structures of wheat, rice, and barley proteins (Figure 4, Figure 5 and Figure 6, Table A1, Table A2, Table A3, Table A4, Table A5, Table A6, Table A7, Table A8, Table A9, Table A10, Table A11, Table A12 and Table A13). The BIOPEP-UWM database program revealed fragments of the LLEY sequence with AntiV activity only in garden pea proteins. Fragments of the ERF sequence were found in wheat protein (one sequence) and also rice, buckwheat, and garden pea proteins, whereas fragments of the VVV sequence with AntiC activity were absent in oat, sorghum, bean, white lupine, sesame, cocoa, ginkgo, pumpkin, and upland cotton proteins (Figure 4, Figure 5 and Figure 6, Table A1, Table A2, Table A3, Table A4, Table A5, Table A6, Table A7, Table A8, Table A9, Table A10, Table A11, Table A12 and Table A13).

### 2.2. Characterization of Amino Acid Composition of Peptides Exhibiting ImmD or ImmS Activity

**Figure 7:**
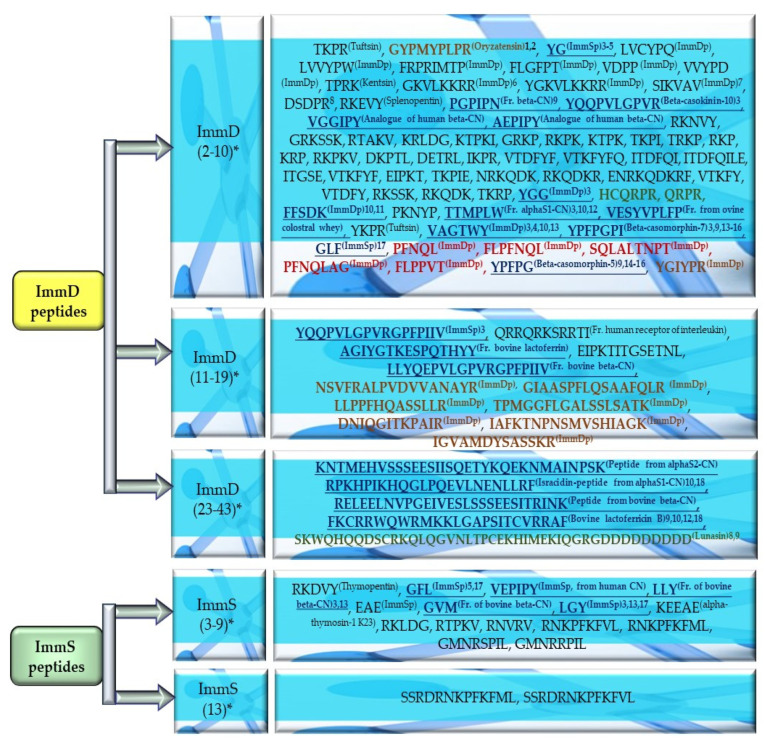
Grouped sequences of ImmD and ImmS peptides originating from the “Bioactive peptides” database (one of the repositories of the BIOPEP-UWM database) [16,29,30,31,32,33,34,35,36,37,38,39,40,41,42,43,44,45,46,47,48,49,50,51,52,53,54,55,56,57,58,59,60,61,62,63]. *—Numbers in brackets denote the number of amino acid residues occurring in molecules of the grouped ImmD and ImmS peptides. The bold font in the **blue**, **red**, **brown**, or **green** colors denotes sequences of biopeptides derived from **milk proteins**, **zein hydrolysates, rice**, and **soybeans**, respectively. The name in superscripts indicates the name of the peptide. The numbers in superscripts indicate the type of activity/peptide: ^1^—opioid antagonist, ^2^—smooth muscle-contracting peptide, ^3^—ACEi-angiotensin-1-converting enzyme inhibitor, ^4^—DPPIVi-dipeptidyl peptidase IV inhibitor, ^5^—DPPIIIi-dipeptidyl peptidase III inhibitor, ^6^—ACTH (adrenocorticotropic hormone) receptor antagonist, ^7^—angiogenesis-stimulating peptide, ^8^—anti-inflammatory peptide, ^9^—anticarcinogenic peptide, ^10^—antibacterial peptide, ^11^—cytotoxic peptide, ^12^—antifungal peptide, ^13^—antioxidative peptide, ^14^—AChEi—acetylcholinesterse inhibitor, ^15^—BChEi—butyrylcholinesterase inhibitor, ^16^—opioid agonist, ^17^—peptide-regulating phosphoinositol metabolism, ^18^—antiviral peptide. ImmDp—immunomodulating peptide, ImmSp—immunostimulating peptide, CN—casein, fr—fragment.

The highest numbers of fragments with ImmD and ImmS activity were identified in the analyzed plant proteins via the BIOPEP-UWM database programs; therefore, the peptides featuring these activities and deposited in this database were also studied for their amino acid composition. The amino acid sequences of biopeptides available in the “Bioactive peptides” database (accessed on 1 October 2023) enabled a computation of the number of amino acid residues and the percentage content of individual molecules of amino acids occurring in their structure. The respective results are presented in Table 1, Table 2, Table 3 and Table 4 and Figure 7. The number of peptides exhibiting ImmD and ImmS activities was 86 and 17, respectively. They were afterward grouped in terms of the number of amino acid residues in their structures (Table 1, Table 2, Table 3 and Table 4, Figure 7). Thus, 80.2% and 88% of the analyzed ImmD and ImmS peptides possessed from 2 to 10 amino acid molecules, respectively (Figure 7). In turn, 11–20 amino acid peptides accounted for 14% and 11.8% of the identified peptides with ImmD and ImmS activities, respectively, whereas only 5.8% of the biopeptides with the ImmD activity possessed >20 amino acids (Table 1, Table 2, Table 3 and Table 4). Some of the analyzed peptides were multi-functional, like the ImmD peptide with the YG sequence, derived from milk proteins (Figure 7). The peptides isolated from milk proteins also contained other ImmD and ImmS peptides, including, i.e., beta-casokinin-10, beta-casomorphin-7, beta-casomorphin-5, isracidin—a peptide derived from alphaS1-casein (CN) (fragment 1–23), peptides from alphaS2-CN, beta-CN, and bovine lactoferricin B (Table 1, Table 2, Table 3 and Table 4).

The structures of ImmD peptides contained mainly the G, Y, lysine (K), proline (P), and arginine (R) residues followed by aspartic acid (D), serine (S), phenylalanine (F), and valine (V), and even up to 33.3% of the isoleucine (I), leucine (L), and threonine (T) molecules (Table 1 and Table 2). In turn, the highest numbers of glutamic acid (E) and L (up to 66.7%), as well as R, V (40%), alanine (A), G, methionine (M), F, P, and Y (up to 33.3%) were identified in the sequences of the ImmS peptides (Table 3 and Table 4).

### 2.3. In Silico Hydrolysis of Plant-Derived Proteins

**Figure 8:**
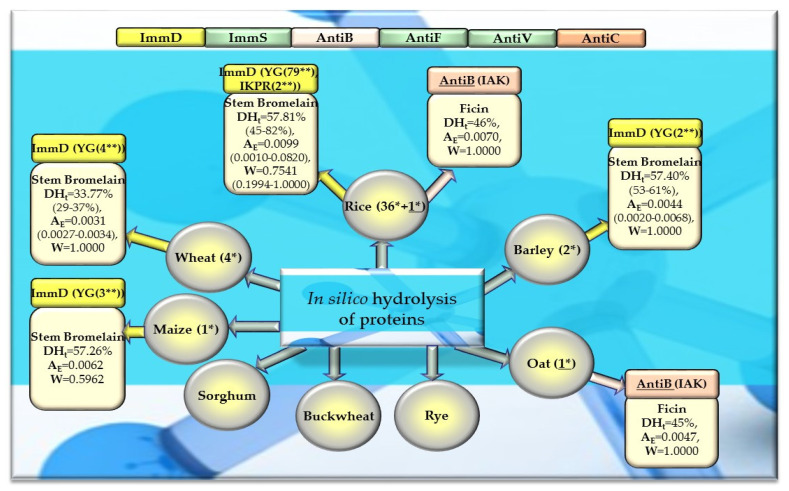
Bioparameters characterizing the in silico hydrolysis of cereal proteins and biosequences (biopeptides) obtained from proteins that potentially modulate the immune system. *—The number of proteins from which biopeptides were released by enzyme (stem bromelain or ficin), **—the number of biopeptides released by the enzyme, ImmD—immunomodulatory, ImmS—immunostimulatory, AntiB—antibacterial, AntiF—antifungal, AntiV—antiviral, AntiC—anticarcinogenic peptides (sequence of biopeptides), amino acid sequence presented in the form of a single-letter amino acid code: A—alanine, R—arginine, G—glycine, I—isoleucine, K—lysine, P—proline, Y—tyrosine), DH_t_—the theoretical degree of hydrolysis (%), mean value (range), A_E_—the parameter A_E_ (the frequency of the release of fragments with a given activity by the selected enzyme), mean value (range), W—the parameter W (the relative frequency of the release of fragments with a given activity by the selected enzyme), mean value (range, defined in the “Materials and Methods” section). This figure was prepared based on the data/results provided in Table 5, Table 6, Table A1, Table A2, Table A3, Table A4 and Table A5.

**Figure 9:**
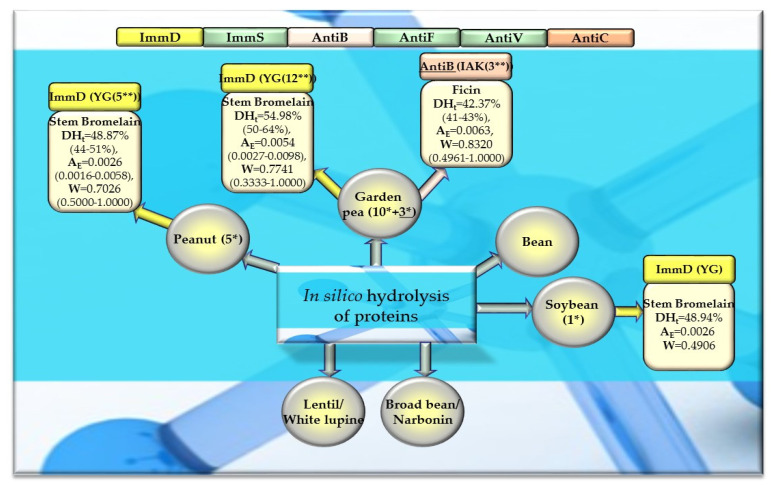
Bioparameters characterizing the in silico hydrolysis of legume proteins and biosequences (biopeptides) obtained from proteins that potentially modulate the immune system. *—The number of proteins from which biopeptides were released by the enzyme (stem bromelain or ficin), **—the number of biopeptides released by the enzyme (stem bromelain or ficin), ImmD—immunomodulatory, ImmS—immunostimulatory, AntiB—antibacterial, AntiF—antifungal, AntiV—antiviral, AntiC—anticarcinogenic peptides (sequence of biopeptides), amino acid sequences presented in the form of a single-letter amino acid code: A—alanine, G—glycine, I—isoleucine, K—lysine, Y—tyrosine), DH_t_—the theoretical degree of hydrolysis (%), mean value (range), A_E_—the parameter A_E_ (the frequency of the release of fragments with a given activity by the selected enzyme), mean value (range), W—the parameter W (the relative frequency of the release of fragments with a given activity by the selected enzyme), mean value (range) (defined in the “Materials and Methods” section). This figure was prepared based on the data/results provided in Table 7, Table 8, Table A6, Table A7, Table A8, Table A9 and Table A10.

**Figure 10:**
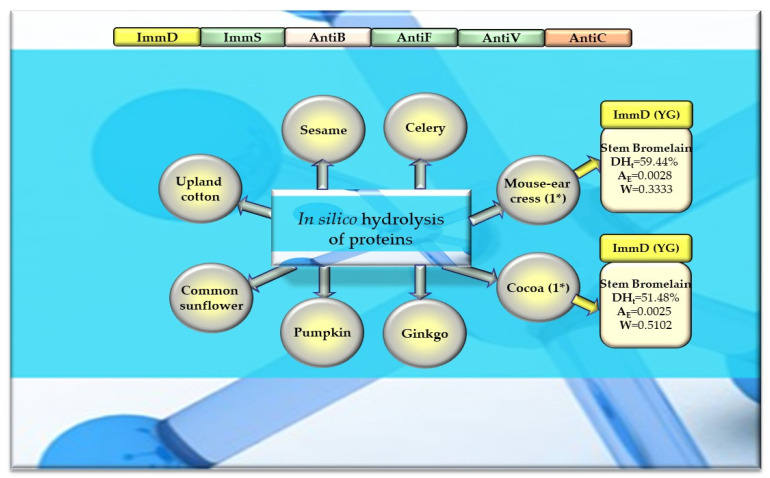
Bioparameters characterizing the in silico hydrolysis of other plant proteins and biosequences (biopeptides) obtained from proteins that potentially modulate the immune system. *—The number of proteins from which biopeptides were released by the enzyme, ImmD—immunomodulatory, ImmS—immunostimulatory, AntiB—antibacterial, AntiF—antifungal, AntiV—antiviral, AntiC—anticarcinogenic peptides (sequence of biopeptides), amino acid sequences presented in the form of a single-letter amino acid code: G—glycine, Y—tyrosine, DH_t_—the theoretical degree of hydrolysis (%), A_E_—the parameter A_E_ (the frequency of the release of fragments with a given activity by the selected enzyme), W—the parameter W (the relative frequency of the release of fragments with a given activity by the selected enzyme, defined in the “Materials and Methods” Section). This figure was prepared based on the data/results provided in Table 8, Table A11, Table A12 and Table A13.

After monocatalytic simulation of the hydrolysis of the analyzed plant proteins, the parameters characterizing this process (DH_t_, A_E_ and W) performed with ficin or stem bromelain or pepsin (pH > 2) were calculated, and sequences of peptides with potential activities modulating the immune system were searched for (ImmD, ImmS, AntiB, AntiF, AntiV, AntiC). Among the analyzed cereal, legume, and other proteins, only stem bromelain released peptides with ImmD activity and ficin with AntiB activity (Table 5, Table 6, Table 7 and Table 8, Figure 8, Figure 9 and Figure 10). In turn, the highest number of immunopeptides with YG sequences was released by stem bromelain as a result of in silico hydrolysis of rice proteins. This enzyme hydrolyzed from 45% to 82% (mean DH_t_ = 57.81%) of the peptide bonds of these proteins and released 79 YG molecules and 2 IKPR molecules from the monomers of the analyzed rice proteins (Table 6, Figure 8). The frequency and relative frequency of release of the fragments with ImmD activity (immunopeptides) by stem bromelain were within the ranges of A_E_ = 0.0010–0.0820 and W = 0.1994–1.000, respectively. The YG dipeptides were also among the hydrolysates of gliadins, glutenins (wheat), hordeins (barley), legumin 1 (maize), garden pea, soybean, peanut, and also mouse-ear cress and cocoa storage protein (Table 5, Table 6, Table 7 and Table 8, Figure 8, Figure 9 and Figure 10).

In turn, ficin—another proteolytic enzyme used for simulated hydrolysis of peptide bonds—released antibacterial IAK peptides from monomers of rice, oat, and garden peas, and the DH_t_ determined for these proteins ranged from 41 to 46% (Table 5, Table 6, Table 7 and Table 8, Figure 8, Figure 9 and Figure 10).

## 3. Discussion

### 3.1. Plant Proteins with Potential Activities Modulating the Immune System

Among the analyzed plant-derived proteins, the most represented ones were those of cereals and legumes, i.e., wheat—190, rice—112, barley—59, and garden pea—53 (Figure 1, Figure 2, Figure 3, Figure 4, Figure 5 and Figure 6, Table A1, Table A2, Table A3, Table A4, Table A5, Table A6, Table A7, Table A8, Table A9, Table A10, Table A11, Table A12 and Table A13). The analyzed proteins belonged to the group of proteins found in crops which are hypothesized to exhibit a lesser autoimmune potential compared to the proteins of the analyzed animal and fish species due to the lower value of their Gershteyn–Ferreira index (GF index). Food-derived proteins are also one of the major components of a human diet, while the latter is deemed to be one of the environmental triggers of certain autoimmune diseases. In turn, molecular mimicry has been found to be one of the mechanisms driving the development of autoimmunization, an inducer of autoimmune diseases. Hence, there is a hypothesis that selected species of animals and plants consumed by humans may promote the development of certain autoimmune diseases. The mapping of the similarity of epitopes between commonly consumed animal species (cow, sheep, goat, chicken, turkey, duck), fish species (tilapia, salmon), plant species (rice, quinoa, soybean, rye, wheat) and various human autoimmune diseases enabled the development of the GF index (its values oscillate around 0–1). The values of this unique autoimmune index allow us to make inferences regarding autoimmunization probability. The analyzed crops had significantly lower GF values compared to those of the investigated animal and fish species [6,27]. However, the cited investigations did not take into account of gliadin peptides responsible for celiac disease development or the citrulline proteins or cyclic peptides that intensify symptoms of rheumatoid arthritis (RS) and SM, respectively [6].

Proteins, including the food-derived ones, are also potential compounds from which fragments of a protein sequence (peptides) may be released, and some of the released peptides may exhibit bioactivity [12,18,19,20,21,22,25]. These bioactive peptides include peptide immunomodulators, which may modulate the human immune response, activate macrophages, stimulate phagocytosis [12,14,15,16,17,18], and contribute to an increased number of leukocytes or immunomodulators (e.g., cytokines, nitric oxide, immunoglobulins) [12,14,15]. The ImmD peptides are one of the most complex groups of BPs and are often multifunctional [6]. Hence, the analyzed plant proteins were determined not only for their ImmD and ImmS activities but also for their AntiB, AntiF, AntiV, and AntiC activities (Figure 1, Figure 2, Figure 3, Figure 4, Figure 5 and Figure 6, Table A1, Table A2, Table A3, Table A4, Table A5, Table A6, Table A7, Table A8, Table A9, Table A10, Table A11, Table A12 and Table A13). The cereal proteins analyzed in this study have already been investigated, but only in terms of their antioxidative activity [64].

The proteins of cereal grains may account for 6 to even 20% of the grain weight [65,66,67], as the protein content of wheat grain reaches 10–15% [68] compared to that found in legume seeds and ranging from 13 to 30 g/100 g dry matter [69,70]. Due to their solubility in water, salt solutions, alcohols, acids, and bases, proteins are divided into albumins, globulins, prolamins, and glutelins, respectively [67,69,71]. Prolamins and globulins are the major proteins of cereal grains. Prolamins of wheat, rye, barley, and sorghum account for 30–50% of total proteins and are called gliadins, secalins, hordeins, and kafirins, depending on the cereal type [71,72,73]. In oat and rice grains, prolamins account for ca. 4–15% [65,66]. In turn, albumins (accounting for 10–20%) and globulins (70%) are the major proteins of peas and other legumes, whereas the globulin fraction is mainly composed of legumins (11S globulin) and vicilins (7S globulin) [69,74], which may be separated by means of two-dimensional gel electrophoresis (2-DE) [74]. Likewise, the cereal proteins may be separated using 2-DE [75] or 1-DE (sodium dodecyl sulfate–polyacrylamide gel electrophoresis, SDS-PAGE*)* [68].

The analyzed wheat, rice, buckwheat, and garden pea proteins turned out to be precursors of peptides with a potential activity affecting the human immune system because they contained fragments of amino acid sequences exhibiting the following activities: ImmD, ImmS, AntiB, AntiV, and AntiC (Figure 1, Figure 2, Figure 3, Figure 4, Figure 5 and Figure 6, Table A1, Table A2, Table A3, Table A4, Table A5, Table A6, Table A7, Table A8, Table A9, Table A10, Table A11, Table A12 and Table A13). However, their structures, and likewise those of other plant-derived proteins, were devoid of fragments with AntiF activity (the adopted criteria for bioinformatic analyses). Among the analyzed cereal proteins, the best precursors of peptides the ImmD activity turned out to be rice proteins, the best of those with the ImmS activity were buckwheat proteins, those with AntiB activity were barley proteins, and those with AntiV activity included a wheat protein (one protein) and buckwheat proteins. The mean values of parameter A computed for these activities reached A = 0.0108, A = 0.0059, A = 0.0060, A = 0.0038 (for one protein of wheat), and A = 0.0035 (for buckwheat proteins). The fragments of amino acid sequences with AntiC activity were most often identified in proteins of wheat, barley, and celery (one protein), whereas the value of parameter A computed for these three groups of proteins reached A = 0.0063 (Figure 1, Figure 2 and Figure 3). The values of parameter A calculated for various activities and for a given protein/proteins enabled an evaluation of this compound/compounds in terms of biological activity (quantitative criterion) and indicating whether the analyzed protein/proteins may be a good source/sources of BPs. However, the values of parameter A made it impossible to simultaneously explain which structural motifs of a protein (fragments of amino acid sequences) are responsible for this activity, or to establish the location of these motifs in the amino acid sequence of a given protein [76]. Therefore, the individual proteins were determined for the profile of their potential bioactivity [28,64,76].

The highest number of fragments with ImmD activity and YG and YGG sequences was identified in rice proteins, i.e., 115 and 17, respectively (Figure 4, Table A2), which was higher compared to proteins of wheat, legumes, and other plant proteins (Figure 5 and Figure 6, Table A1, Table A3, Table A4, Table A5, Table A6, Table A7, Table A8, Table A9, Table A10, Table A11, Table A12 and Table A13). The YG is a fragment (a fragment released from a protein structure is a peptide) that enhances protein biosynthesis in lymphocytes. A peptide possessing this sequence is released from milk proteins [31] and was also identified in cod protein hydrolysates [77] and in amaranth protein hydrolysate [78]. YG is a multi-active peptide exhibiting activities of inhibitors (and) enzymes: angiotensin-1-converting enzyme (ACE) (EC 3.4.15.1) (ID 3553) [79], dipeptidyl peptidase III (DPP-III) (EC 3.4.14.4) (ID 9508) [80], and dipeptidyl peptidase IV (DPP-IV) (EC 3.4.14.5) (ID 8936) [81]. The EC_50_ value of YG (ID 3553) computed for the ACEi activity was EC_50_ = 1523.00 µM [79], and for YGG—a fragment of the sequence with ImmD activity which also exhibited ACEi activity—it was EC_50_ = 1001.00 µM [82]. The EC_50_ value is defined as the concentration of a peptide (inhibitor), which causes a 50% decrease in enzyme activity (expressed in millimoles (mM) or micromoles (µM)) under experimental conditions [28,83,84,85].

ACE (EC 3.4.15.1) and DPP-IV (EC 3.4.14.5) are enzymes which play a key role in blood pressure regulation and degradation of intestinal hormones (incretins), respectively [81,83,84,85,86,87]. The first catalyzes the hydrolysis of angiotensin-1 to angiotensin-2 and activates bradykinin, consequently leading to blood pressure increases [18,83,84]. The ACE inhibitors decrease blood pressure by inhibiting the activity of angiotensin-converting enzyme; hence, the peptide inhibitors of ACE act similarly to many hypotensive drugs, e.g., captopril, enalapril, cilazapril, or lisinopril [84,86]. The second enzyme, DPP-IV, is a serine protease occurring in human tissues and organs, for which enteral hormones (incretins) serve as substrates. Such incretins include glucagon-like peptide-1 (GLP-1) and glucose-dependent insulinotropic polypeptide (GIP), which are degraded by DPP-IV. GLP-1 stimulates insulin secretion from pancreatic beta cells in a glucose concentration-dependent manner [81,83,84,85,86]. Its stabilization by controlling DPP-IV activity promotes insulin secretion, leading to decreased blood glucose levels and thereby affecting the control of glycemia (an indicator of blood glucose levels) in patients with type 2 diabetes. The DPP-IV inhibitors, e.g., sitagliptin, vildagliptin, and saxagliptin, are often used in the pharmacotherapy of this type of diabetes [81,84,85,86].

Fragments of the YG sequence with ImmD activity were also detected in other proteins, i.e., 33 and 19 fragments possessing this sequence were identified in garden pea and barley proteins, respectively, whereas 16, 8, and 5 fragments were identified in the YG sequence in wheat, peanut, and maize proteins, respectively (Figure 4, Figure 5 and Figure 6, Table A3, Table A4, Table A5, Table A6, Table A7, Table A8, Table A9, Table A10, Table A11, Table A12 and Table A13). The highest number of fragments of the GLF sequence with ImmD activity was determined in the proteins of rice—11 and garden peas—7 (Figure 4, Figure 5 and Figure 6, Table A1, Table A2, Table A3, Table A4, Table A5, Table A6, Table A7, Table A8, Table A9, Table A10, Table A11, Table A12 and Table A13). A peptide possessing the GLF sequence with ImmD activity was earlier isolated from human and bovine alpha-lactalbumin (alpha-la) [50]. Fragments of the YG and GLF sequences were detected in all 69 peptides exhibiting ImmD activity, which contained up to 10 amino acid residues and were deposited in one of the BIOPEP-UWM repositories (in the “Bioactive peptides” database) (Table 1, Table 2, Table 3 and Table 4, Figure 7). GLF was also a peptide regulating phosphoinositol metabolism (ID 2740), and the highest number of fragments possessing this sequence was found in proteins of rice and garden peas (Figure 4, Figure 5 and Figure 6, Table A1, Table A2, Table A3, Table A4, Table A5, Table A6, Table A7, Table A8, Table A9, Table A10, Table A11, Table A12 and Table A13). As shown via in vitro analyses, both the natural and synthetic GLF peptides exhibited activity that stimulated phagocytosis of sheep red blood cells (SRBCs) by mouse peritoneal macrophages and protected the mice against infections induced by *Kl. pneumoniae* (dose of 1 mg/kg body weight) [50].

The ProParam tool was used to compute the amino acid composition (number and % content of individual amino acid residues in peptide structures) of peptides exhibiting ImmD and ImmS activities because the fragments of sequences with these activities prevailed in the analyzed proteins (Figure 4, Figure 5, Figure 6 and Figure 7, Table A1, Table A2, Table A3, Table A4, Table A5, Table A6, Table A7, Table A8, Table A9, Table A10, Table A11, Table A12 and Table A13) [88]. ProParam is a tool which, based on the amino acid sequences, also enables computing of other physicochemical parameters, like the molecular weight, theoretical pI, atomic composition, extinction coefficient, estimated half-life, instability index, aliphatic index, and grand average of hydropathicity (GRAVY). However, it proves unsuitable for the analysis of compounds containing <5 amino acid residues; therefore, their amino acid composition was calculated manually [89,90].

The peptides with ImmD activity and possessing up to 10 amino acid residues in their structure that were deposited in the “Bioactive peptide” database, represented the most numerous group of bioactive fragments identified in the analyzed proteins. Proteins of rice were found to contain a nine-amino acid fragment with ImmD activity called oryzatensin (GYPMYPLPR). This fragment was derived from both rice protein and the isolated guinea pig ileum (ID 2840) (Figure 4 and Figure 7) [30]. The GYPMYPLPR (oryzatensin) fragment was also identified in rice protein as an allergenic protein (japonica cultivar-group) (Figure 4, Table A2). This ImmD peptide has been reported to stimulate phagocytosis of human multi-loci leukocytes and promote the generation of superoxide anions by human peripheral leukocytes. Oryzatensin is also an opioid antagonist (ID 2839) [30] and smooth muscle-contracting peptide (ID 2838) [91].

The rice proteins were also identified to contain fragments of a 6-amino acid sequence (YGIYPR) and 3 16–17-amino acid peptides typical only of this group of the analyzed cereal proteins. This is because they were isolated only from rice proteins and were identified by the BIOPEP-UWM database programs only in this groups of proteins [51] (Figure 4, Figure 5 and Figure 6, Table A1, Table A2, Table A3, Table A4, Table A5, Table A6, Table A7, Table A8, Table A9, Table A10, Table A11, Table A12 and Table A13). The YGIYPR peptide was isolated from the trypsin hydrolysates of rice proteins, which were purified and then identified using high-performance liquid chromatography (HPLC) and electrospray ionization quadrupole time-of-flight (ESI-QTOF) mass spectrometry. The YGIYPR turned out to be an active peptide exhibiting proliferation of RAW 264.7 macrophage cell line in the range of 12.5–100 µg/mL [51]. In turn, the 12–17-amino acid peptides (DNIQGITKPAIR, IAFKTNPNSMVSHIAGK and IGVAMDYSASSKR) derived from rice proteins inhibited the production of nitric oxide and proinflammatory cytokines (interleukin-1-beta, interleukin-6 (IL-6) and tumor necrosis factor-alpha (TNF-alpha)) by RAW 264.7 mice macrophages stimulated by lipopolysaccharides (LPSs) [56]. One of the aforementioned fragments of the IAFKTNPNSMVSHIAGK sequence was detected in the analyzed rice proteins (glutenin) (Table A2), whereas fragments of the NSVFRALPVDVVANAYR and GIAASPFLQSAAFQLR sequences were identified in rice glutelins and prolamins (clone PPROL 7, 14, 4A), respectively (Table A2). They inhibited NO production at concentrations of 6.25 μg/mL and 12.5 μg/mL, respectively. In turn, the lowest TNF-alpha concentrations were determined at a peptide concentration of 6.25 μg/mL (for two peptides). The lowest IL-6 levels were assayed at peptide concentrations of 12.5 μg/mL and 3.125 μg/mL, whereas IL-1-beta inhibition was assayed at peptide concentrations of 3.125 μg/mL and 6.25 μg/mL [55].

Macrophages constitute the first line of host defense against infections, and when stimulated by LPS, they produce pro-inflammatory cytokines including IL-1, IL-6, and TNF-alpha [55]. The innate response may be triggered by the activation of such PRRs as Toll-like receptors (TLR). PRRs may be bound by pathogen (or damage)-associated molecular patterns (PAMPs, DAMPs), e.g., lipopolysaccharide components of cell walls of Gram-positive and Gram-negative bacteria or two-stranded RNAs—molecules typical of pathogens (absent in the cells of mammals). In turn, the DAMPs included the following molecules: high mobility group box 1 (HMGB1), DNA, RNA, and S100. The activation of PRRs leads to the release of inflammatory cytokines, resulting in the aging of dendritic cells and presentation of antigens to the adaptive immune system [2].

The BIOPEP-UWM database program identified a fragment of the PFNQL sequence with ImmD activity in the structure of one sorghum prolamin (kafirin PSKR2 precursor, sorghum bicolor, ID 1196) (Figure 4, Table A5). A peptide possessing the PFNQL sequence was also isolated from hydrolysates of the major maize protein, i.e., from zein hydrolysates. Zein is the main protein of maize, which is rich in glutamic acid, leucine, and proline but deficient in lysine and tryptophan [16]. One of the fractions of the termolysin hydrolysates of zein was identified using liquid chromatography–tandem mass spectrometry (LC-MS/MS) to contain five peptides with PFNQL, FLPFNQL, SQLALTNPT, GAPFNQL, and FLPPVT sequences. These sequence fragments (peptides) were released from alpha-zein. The FLPFNQL peptide exhibited the highest capability for inhibiting IL-6 production (57.7%  ±  6.1%) at a concentration of 0.5 mM. In turn, PFNQL caused 46.2%  ±  4.5% inhibition of IL-6 generation [16].

The human monocyte cell line U937 was used in a model experiment aimed to investigate macrophage functions and immunomodulating potential. IL-6 is a mediator engaged in the regulation of the acute phase response to damages and infections, whereas its dysregulation may result in the development of autoimmune diseases (RHs, MS) [16]. The FLPFNQL and PFNQL peptides were more effective in inhibiting IL-6 generation in the U937 cells compared to the PFNQLAG peptide, and their activity could be affected by fragments of their “PFNQL” amino acid sequence. However, the FLPFNQL peptide found in zein hydrolysates obtained via alcalase hydrolysis exhibited higher activity than the PFNQL peptide, which indicates that the location of FL amino acid residues, especially at the N-terminus of a peptide molecule, could determine its higher immunomodulating activity. In turn, the amino acid residue Q is, most likely, an important immunological component used by immune cells, which may contribute to the higher anti-inflammatory activity of FLPFNQ [16]. This means that the bioactivity of peptides (their interaction with body receptors to induce a specified biological effect) [18] correlates, most of all, with their chemical structure, with key roles attributed to the amino acid sequence; type, hydrophobic properties, and presence of polar amino acids in their structure; as well as spatial arrangement of amino acid molecules [14,16,18,19,20,21,23,24,26].

The peptide sequences with ImmD activity derived from zein hydrolysates accounted for 5.8% of the “Bioactive peptides” database resources (one of the BIOPEP-UWM repositories). A total of 10.5% of the biopeptides exhibiting this activity were derived from rice proteins, whereas 23.3% and 29.4% of the deposited biopeptide sequences with ImmD and ImmS activities, respectively, were isolated from milk proteins (Table 1, Table 2, Table 3 and Table 4, Figure 7).

Fragments of the amino acid sequences with ImmS activity were identified in 38 and 20 analyzed proteins of rice and garden peas, respectively (mean A = 0.0044 and A = 0.0048, respectively) (Figure 1 and Figure 2) and also in three proteins of buckwheat and broad beans (mean A = 0.0059 and A = 0.0053) (Figure 1 and Figure 2). The fragments of sequences with ImmS activity were also detected in one protein of mouse-ear cress, white lupine, and celery, but the A values determined for the three single amino acid sequences of these proteins were the highest, reaching A = 0.0076 and A = 0.0063 for white lupine and celery, respectively. This means that the frequency of ImmS fragment occurrence in these proteins was higher compared to the analyzed proteins of rice and garden peas (Figure 1, Figure 2 and Figure 3). In turn, the highest number of GFL, LGY, EAE, GVM and LLY sequences with ImmS activity was detected in cereal proteins (rice and barley), followed by the EAE, LLY, GVM, and GFL sequences detected in garden pea proteins as well as the LLY and EAE sequences found in broad bean proteins (Figure 4, Figure 5 and Figure 6, Table A1, Table A2, Table A3, Table A4, Table A5, Table A6, Table A7, Table A8, Table A9, Table A10, Table A11, Table A12 and Table A13). The fragments of amino acid sequences LLY and LGY are the fragments released from milk proteins, exhibiting not only ImmS activity [50,61] but also ACEi activity (ID(s) 10167, 9284) [92,93] and antioxidative activity (ID(s) 10168, 10060) [92,94]. The LGY sequence is also a peptide sequence deposited in the “Bioactive peptides” database as a peptide regulating phosphoinositol metabolism (ID 2738) (Figure 7) [95].

In the in vitro tests, the LLY fragment (released from cow casein) exhibited activity-stimulating phagocytosis of sheep red blood cells (SRBCs) by mouse peritoneal macrophages and secretion of antibodies against SRBCs by murine spleen cells, but it did not protect the mice against infections induced by *Kl. pneumoniae* like the GLF peptide did (peptide with ImmD activity) [50].

The highest number of IQY sequences with AntiB activity was determined in the primary structures of wheat, rice, and barley proteins, whereas that of the YVL sequences in the same cereal proteins were also in buckwheat, garden pea, broad bean, white lupine, and mouse-ear cress proteins. In turn, the highest number of IAK sequences was found in the structures of rice and garden pea proteins (Figure 4, Figure 5 and Figure 6, Table A1, Table A2, Table A3, Table A4, Table A5, Table A6, Table A7, Table A8, Table A9, Table A10, Table A11, Table A12 and Table A13). Fragments of the LLEY sequence with AntiV activity [96] were detected only in garden pea proteins, and those of the ERF sequence were found in proteins of wheat (one sequence), rice, buckwheat, and garden peas (Figure 4, Figure 5 and Figure 6, Table A1, Table A2, Table A3, Table A4, Table A5, Table A6, Table A7, Table A8, Table A9, Table A10, Table A11, Table A12 and Table A13). The fragment of sequence (ERF) was multi-active (AntiV and antioxidant activity) [23,88]. Furthermore, the highest number of VVV fragments with the AntiC activity was found in wheat proteins (Figure 4, Figure 5 and Figure 6, Table A1, Table A2, Table A3, Table A4, Table A5, Table A6, Table A7, Table A8, Table A9, Table A10, Table A11, Table A12 and Table A13). The fragments of the YVL (ID 8268) and IAK (ID 10105) sequences exhibited AntiB activity and were isolated from milk proteins, whereas peptides were released from kappa-casein by pepsin and showed activity mainly against Gram-positive bacteria [97]. The second fragment was released from the same protein, but by plasmin [98]. Both fragments of amino acid sequences were multi-active, but YVL also exhibited antioxidative activity (ID 8150) [99], whereas IAK additionally affected the cardiovascular system as it was deposited as both ACEi (EC_50_ = 15.70 µM, ID 7626) [100] and hypotensive peptide (ID 10106) [101].

### 3.2. Bioevaluation of In Silico Hydrolysis of Plant Proteins and Enzymatic Preparations with Potential Properties Modulating the Immune System

In order to release bioactive fragments (biopeptides) from the primary structures of the analyzed plant-derived proteins, monocatalytic simulations of hydrolysis of peptide bonds were performed, exploiting the specificity of action of three selected enzymes and tools available in the BIOPEP-UWM database (“Enzyme(s) action” app). This part of the BIOPEP-UWM database contained 33 various proteolytic enzymes [88]. Two phytoenzymes, ficin (EC 3.4.22.3) and stem bromelain (EC 3.4.22.32), and one digestive enzyme, pepsin (pH > 2) (EC 3.4.23.1) were used in the present study. Ficin, stem bromelain, and pepsin (pH > 2) were hydrolyzed peptide bonds containing F-, G-, and L-residues from the side of the carbonyl group occurring in the peptide bond (the so-called C-terminus). In addition, ficin also forms select bonds with K-, V-, S-, R-, and H-residues and hydrolyzed them also from the C-terminal side of the peptide bond. Additionally, stem bromelain searched for V-, A-, T-, R-, and S- residues in the peptide bonds and cleaved them from the same side as ficin did. In turn, pepsin (pH > 2) searched for Y-, A-, E-, Q-, T-, N-, K-, D-, and M- residues and hydrolyzed the peptide bonds containing them from the C-terminus side, and also for V- and I-residues and hydrolyzed them from the N-terminus side [88].

The simulated hydrolysis of peptide bonds was performed using the specificity of action of the same enzymes as those used in earlier studies to release antioxidative peptides from protein structures [64]. The proteolytic enzymes selected for this study are also applied in the food industry for, e.g., meat tenderization, whereas pepsin is a digestive enzyme that is also synthesized in the gastrointestinal tract [102,103,104]. Due to their bioactivity and not only catalytic activity, these enzymes can be classified as nutraceuticals [105,106,107].

Stem bromelain exhibits a broad spectrum of biological activities, as it has been reported to affect the cardiovascular system, blood coagulation, and fibrinolysis, as well as to exhibit analgesic activity by influencing pain mediators (such as bradykinin) and anticarcinogenic activity [108]. In the in vitro analyses, bromelain was shown to be capable of modulating surface adhesion molecules on T cells, macrophages, and NT cells. It also induced the secretion of IL-1-beta, IL-6, and TNF-alpha by peripheral blood mononuclear cells (PBMCs), blocked the Raf-1/extracellular-regulated kinase (ERK) 2 pathways, and suppressed the activation of CD4+ T cells and the expression of CD25. Furthermore, it decreased the level of a transcription factor playing a key role in the regulation of immune responses to infections, i.e., the nuclear factor kappa-light-chain-enhancer of activated B cells (NF-kappaB) and cyclooxygenase (Cox-2) expression in mouse papillomas and in models of skin tumourigenesis [108].

Cox-2 is an enzyme which catalyzes arachidonic acid conversion to prostaglandin-E_2_ (PGE2) and serves an important biological role similar to NF-kappaB and PGE2, promoting angiogenesis and cancer progression [109]. Bromelain inhibited bacterial endotoxin (LPS)-induced NF-kappaB activity and the expression of PGE2 and Cox-2 in human monocytic leukemia and murine microglial cell lines. It also promoted apoptopic cell death in neoplasms by suppressing the activity of cell survival regulators such as (ERK) and (Akt) [108].

Out of the three enzymes (ficin, stem bromelain, pepsin (pH > 2)) used in the present study for plant protein hydrolysis simulation, only stem bromelain and ficin released bioactive peptides (with ImmD and AntiB activities, respectively) (Table 5, Table 6, Table 7 and Table 8, Figure 8, Figure 9 and Figure 10). The bromelain hydrolysates contained immunopeptides with YG and IKPR sequences. In addition, only stem bromelain released ImmD peptides with the YG sequence, and the highest number of these peptides (81 molecules) was released from rice proteins, including two possessing the IKPR sequence (Table 5, Table 6, Table 7 and Table 8, Figure 8, Figure 9 and Figure 10). A total of 68.7% of the fragments possessing the YG sequence and exhibiting ImmD activity, which were identified in rice proteins, were released as immunopeptides (Table A2 and Table 6, Figure 4 and Figure 8). The YG immunopeptides were also released from wheat, maize, barley, garden pea, and peanut proteins, i.e., 4, 3, 2, 12, and 5 molecules, respectively, as well as from soybean, mouse-ear cress, and cocoa proteins (one molecule from each) (Figure 8, Figure 9 and Figure 10), but only the parameter W computed for wheat and barley proteins reached W = 1.0000 (Table 5, Table 6, Table 7 and Table 8, Figure 8, Figure 9 and Figure 10). The parameter W was one of the parameters characterizing in silico proteolysis (protein hydrolysis simulation) and was defined as the relative frequency of release of fragments with a given activity by selected enzymes (see “Materials and Methods” section) [28,88]. The W = 1.0000 calculated for wheat and barley proteins indicated that all fragments possessing the sequence with ImmD activity that were found in the sequence of the analyzed wheat and barley protein (only protein groups) (Table A1 and Table A3) in the BIOPEP-UWM database programs were released from these sequences as ImmD peptides upon simulated peptide bond hydrolysis by stem bromelain (Table A1, Table A3 and Table 5, Figure 8).

The YG peptides, similar to the YGG ones, were released from milk proteins (from kappa-casein and alpha-la, respectively) and modulated the proliferation of human peripheral blood lymphocytes (PBLs) [31]. The PBLs are mature lymphocytes and one of the few types of white blood cells (WBCs) that are of key importance to the immune system (they include B, T, and NK cells) and cooperate with each other to protect the body against bacteria, viruses, and other pathogenic toxins [110]. Changes triggered in PBL proliferation by YG and YGG were investigated after 5-bromo-2′-deoxyuridine (BrdU) incorporation into DNA, whereas the impact of bioactive peptides on the protein biosynthesis of PBLs was determined based on the [^3^H] leucine incorporation test. The YG dipeptide caused a significant increase (maximally by 90%) in human PBL proliferation and was a more active biopeptide compared to the analyzed YGG peptide, which promoted PBL proliferation by up to 35%. Both YG and YGG significantly enhanced the incorporation of BrdU by PBLs at peptide concentrations ranging from 10^−11^ to 10^−4^ M/L, but their maximal stimulating effect was observed at concentrations of 10^−4^ and 10^−8^ M/L, respectively. These peptides have been applied in the immunotherapy of human immunodeficiency virus infection. Di- and tripeptides like YG and YGG may interact with the gut-associated lymphoid tissue (GALT), transit through the intestine, and reach peripheral lymphocytes [31]. YG is not only a peptide exhibiting ImmD activity but also a multi-active peptide showing, e.g., antihypertensive activity (ID 3553) [79,88], as it inhibited angiotensin-1 hydrolysis to angiotensin-2 (inhibitor of angiotensin-1-converting enzyme (ACE) (EC 3.4.15.1)). The conversion of a decapeptide, i.e., angiotensin-1 (DRVYIHPFHL) to an octapeptide, namely angiotensin-2 (DRVYIHPF), causes vasoconstriction; hence, ACE activity inhibition causes blood pressure reduction. The concentration of this peptide inhibitor triggering an enzyme activity reduction of 50% (EC_50_) was reported to reach 1523.00 µM [79] and 2000 µM [88]. The YG biopeptide (YG) is known as alpha-lactokinin because it was isolated from alpha-la (fragments 18–19, 50–51) and kappa-casein (fragment 38–39) [88]. It was also identified in trypsin-digested amaranth glutelins via mass spectrometry [78] and in cod protein hydrolysates [77]. YG is also an inhibitor (i) of DPP-IV (ID 8936) [81,88] and DPP-IIIi (ID 9508) [80,88]. DPP-IVi prevents the degradation of intestinal hormones (GLP-1 and GIP) [81,83,84,85,86], whereas DPP-IIIi inhibits the hydrolysis of a neurotransmitter found in the brain and interacting with morphine receptors, thereby relieving pain [80].

Dipeptidyl peptidase-III (DPP-III; EC 3.4.14.4) is a proteolytic enzyme which catalyzes hydrolytic cleavage of dipeptides from peptide substrates (from the N-terminal side). It was isolated from extracts of the bovine pituitary gland and showed relatively high specificity against a series of peptides, i.e., from tetrapeptides to oktapeptides [111]. DPP-III exhibits a high affinity to bioactive peptides, anigotensins (angiotensin-2, angiotensin-3), enkephalins, and endomorphins. It hydrolyzes pentapeptides and Leu- and Met-enkephalin at the Gly2–Gly3 bond as the so-called enkephalinase B [80,111]. Leu-enkephalin is an important brain neurotransmitter that interacts with morphine receptors and reduces pain. The products of Leu-enkephalin hydrolysis included YG and GFL, and the latter inhibited DPP-III activity (80% inhibition at 1.0 mM concentration) in the non-competitive mode (non-competitive inhibition), which may indicate that GFL can be one of the endogenous mechanisms of DPP-III activity regulation. The value of the inhibition constant K_i_ reached 15 mM [80]. The increased concentration of DPP-III was observed in endometrial and ovarian malignancies, whereas its overexpression was correlated with a poor prognosis for human breast cancer and colorectal cancer patients. In turn, the elevated concentration of this enzyme was determined in the plasma of patients with sepsis [111].

Even though GFL as a fragment of amino acid sequences with ImmS activity was detected in sequences of the analyzed proteins (rice, wheat, barley, garden pea, bean, soybean, sesame, celery, and mouse-ear cress) (Figure 4, Figure 5 and Figure 6), it was not released from their structures by any of the three proteolytic enzymes used for hydrolysis simulation (Table 5, Table 6, Table 7 and Table 8, Figure 8, Figure 9 and Figure 10).

Immunopeptides are peptides with 2 to 20 amino acids residues in their structures, including mainly hydrophobic acids like G, V, L, P, Y and also glutamic acid (E) [15]. G, P, Y, V, and L amino acids were found in the structures of ImmD peptides that were deposited in the BIOPEP-UWM database (Table 1 and Table 2). In turn, the sequences of ImmS peptides deposited in the database were predominated by glutamic acid (E) and L (up to 66.7%), followed by R and V (40%) as well as A, G, M, F, P, and Y (up to 33.3%) (Table 3 and Table 4).

Protein hydrolysates affect the intestinal epithelial cells, intestinal immune cells (including the numbers of IgA^+^ B cells and activation of T cells in the intestine), mesenteric lymph nodes, and the systemic immune system. In the lumen, i.e., in the intestinal space where food bulk transits and undergoes digestion and absorption, the bioactive peptides and protein hydrolysates provided with food or synthesized in the gastrointestinal tract may strengthen the epithelial barrier and production of mucus and antimicrobial proteins which remove pathogens [15]. Di- and tripeptides, which were also identified in the bromelin hydrolysates of plant proteins (Table 5, Table 6, Table 7 and Table 8, Figure 8, Figure 9 and Figure 10) are compounds which, when mediated by the peptide transporter PepT1 found in intestinal epithelial cells, may enter the bloodstream. Larger peptides make use of endocytosis, in which a key role is attributed to hydrophobic interactions between the peptide and the cell membrane by taking part in peptide internalization. The process of endocytosis was also exploited by the immune cells to absorb immunomodulating peptides. One of the immunomodulating peptides was lunasin (a 43-amino acid peptide, SKWQHQQDSCRKQLQGVNLTPCEKHIMEKIQGRGDDDDDDDDD) derived from soybeans, which interacted with the alphaVbeta3 integrin, leading to the inhibition of proinflammatory markers mediated by alphaVbeta3 integrin and to downregulation of the Akt-mediated NF-kappaB pathway [15]. The amino acid sequence of lunasin contained ca. 25% aspartic acid (D) and was deposited in the “Bioactive peptides” database (one of the BIOPEP-UWM repositories) as an immunomodulating peptide (ID 10124) [58], anti-inflammatory peptide (ID 9526) [112], and anticarcinogenic peptide (ID 9525) [113,114]. However, a fragment of the amino acid sequence corresponding to lunasin was not identified in the analyzed plant protein sequences (Table 4, Table 5, Table 6, Table A1, Table A2, Table A3, Table A4, Table A5, Table A6, Table A7, Table A8, Table A9, Table A10, Table A11, Table A12 and Table A13), and such a peptide was not released by the proteolytic enzymes under the experimental conditions of the present study (Table 5, Table 6, Table 7 and Table 8, Figure 8, Figure 9 and Figure 10).

Antimicrobial peptides are long-chain peptides containing from 20 to 50 and even more amino acid residues in their structures. The presence of base amino acids, like K or R, is determined in these peptides by their cationic nature, which, when coupled with the hydrophobic cores, impart the amphipathic character to these peptides and also determine their appropriate spatial structure [14,26]. Ca. 50% of amino acids found in the structures of antimicrobial peptides are residues of the hydrophobic amino acids L, V, F, and Y, which, together with cationic amino acids, determine the antimicrobial properties of these biocompounds [14,115]. Peptides with such unique structures may form channels and/or pores on the surface of microbial membranes, consequently leading to membrane disruption and even to microbial cell division. These positively charged biopeptides interact with negatively charged groups on the surface of microbial membranes and attach to them [14,26]. This process is strongly affected by electrostatic and hydrophobic interactions, enabling further interactions with the lipid components of microbial cell membranes. This leads to membrane disintegration and, ultimately, breakage [14]. Due to the unique structural traits of antimicrobial peptides, including of those deposited in the “Bioactive peptides” repository (BIOPEP-UWM database), only one and three IAK peptides were released from rice/oat and garden pea proteins, respectively, out of all analyzed plant proteins and the fragments with antimicrobial activity (AntiB, AntiF, AntiV). Of these proteins, only ficin released AntiB peptides (Table 5, Table 6, Table 7 and Table 8, Figure 8, Figure 9 and Figure 10). A biopeptide with this sequence was previously identified in plasmin hydrolysates of kappa-casein [98].

## 4. Materials and Methods

### 4.1. Materials

This study was conducted using 524 amino acid sequences of plant-derived proteins (and/or protein fragments) written in the form of single-letter amino acid codes and 4,710 amino acid sequences of biologically active peptides. The sequences were deposited in the BIOPEP-UWM database (https://biochemia.uwm.edu.pl/biopep-uwm/) and derived from database repositories called “Proteins” and “Bioactive peptides” (with 66 different activities) (accessed on 1 October 2023) [88].

The proteins selected for this study originated from wheat (*Triticum aestivum*, *Triticum aestivum* subsp. *Spelta*, *Triticum aestivum* ssp. *sphaerococcum*, *Triticum aestivum* ssp. *compactum*, *Triticum aestivum* spp. *tibeticum*, *Triticum turgidum* subsp. *durum*) (190), rice (*Oryza sativa*, *Oryza sativa* (*japonica cultivar group*), *Oryza sativa* subsp. *japonica*), *Oryza sativa* (*indica cultivar-group*)) (112), barley (*Hordeum vulgare*) (59), oat (*Avena sativa*) (23), buckwheat (*Fagopyrum esculentum*, *Fagopyrum gracilipes*, *Fagopyrum tataricum*) (13), rye (*Secale cereale*) (16), sorghum (*Sorghum vulgare*) (3), maize (*Zea mays*) (2), garden peas (*Pisum sativum*) (53), French/kidney beans (*Phaseolus vulgaris*), runner beans (*Phaseolus coccineus*) (9), soybeans (*Glycine max*) (8), broad beans (*Vicia faba*) (3), Narbon beans (*Vicia narbonensis*, *Vicia pannonica*) (2), peanuts (*Arachis hypogaea*) (7), celery (*Apium graveolens*) (5), sesame (*Sesamum indicum*) (5), and others (14) (lentil, white lupine, mouse-ear cress, cocoa, ginkgo, pumpkin, common sunflower, upland cotton, rape, serendipity berry) [88].

### 4.2. Methods

**Figure 11:**
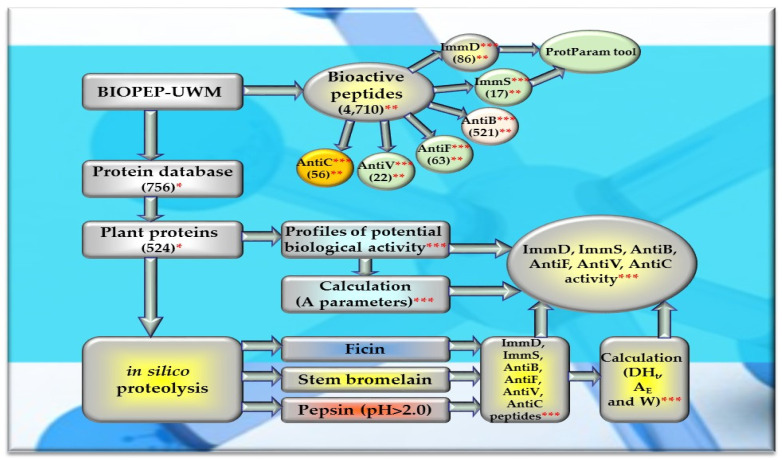
The procedure scheme. *—The number of total proteins including all protein fragments available from the BIOPEP-UWM database, **—the total number of bioactive peptides (accessed on 1 October 2023, https://biochemia.uwm.edu.pl/biopep-uwm/), *** ImmD—immunomodulating/immunomodulatory, ImmS—immunostimulating/immunostimulatory, AntiB—antibacterial, AntiF—antifungal, AntiV—antiviral, AntiC—anticancer/anticarcinogenic activity/peptides.

A profile of the potential bioactivity (considering all fragments of sequences exhibiting various bioactivities found in the analyzed protein) was generated for each analyzed protein/protein fragment to gather data about their 6 activities: immunomodulatory (ImmD), immunostymulatory (ImmS), antibacterial (AntiB), antifungal (AntiF), antiviral (AntiV), and anticarcinogenic (AntiC). The values of parameter A were computed for each activity type, and the percentage content of amino acids was also calculated for the structures of all ImmD and ImmS peptides available in the “Bioactive peptides” database. Monocatalytic simulations of proteolysis (in silico hydrolysis of proteins) were conducted using the selected enzymes (ficin (EC 3.4.22.3), stem bromelain (EC 3.4.22.32), or pepsin (pH > 2) (EC 3.4.23.1)), and the resulting hydrolysates were evaluated for their bioactivities using tools available in the BIOPEP-UWM database [28,88]. Figure 11 depicts the scope of the conducted bioinformatic analyses.

#### 4.2.1. Characterization of Plant-Derived Proteins as Precursors of Peptides with Potential Immune System-Modulating Activities (ImmD, ImmS, AntiB, AntiF, AntiV, AntiC)

##### Profiles of Potential Bioactivity (ImmD, ImmS, AntiB, AntiF, AntiV, AntiC) of the Analyzed Plant-Derived Proteins

A profile of the potential bioactivity was generated for each analyzed protein or a protein fragment considering all types of activities available in the BIOPEP-UWM database repositories throughout the period during which the bioinformatic analyses were conducted (i.e., 2020–2023 and an update as of 1 October 2023) [88].

The activity profile was generated immediately after entering the “Proteins” database (one of the BIOPEP-UWM repositories), opening “Analysis” and “Profiles of potential biological activity” tabs, and choosing “all activities” from the “Select activity” window. A plant protein was selected from the “Protein database” database, and the selected data were accepted. Afterwards, the BIOPEP-UWM database program generated the profile of the potential bioactivity of a given protein based on its amino acid sequence and all sequences of biologically active peptides (4,710 peptides available in the “Bioactive peptides” database). The generated report included the following data: identification number of the bioactive fragment (biopeptide) found in the sequence of the analyzed protein and deposited in the “Bioactive peptides” database (as a biologically active peptide), name of peptide, type of activity, number(s), sequence, and location of the peptide. These data were provided for all the biofragments of the amino acid sequences found in the analyzed protein chain, including their location and activity type [28,88]. Based on the profile generated for each analyzed compound, data were selected regarding the 6 analyzed biological activities: ImmD, ImmS, AntiB, AntiF, AntiV, and AntiC, including the numbers and sequences of amino acids of the biofragments that had been found.

##### Calculation of the Frequency of Bioactive Fragment Occurrence in a Protein Sequence (A) (refers to ImmD, ImmS, AntiB, AntiF, AntiV, and AntiC activities)

The values of parameter A were computed for each analyzed protein (i.e., for the selected protein and for the selected types of bioactivities, following the assumptions of the bioinformatic experiment, Figure 11). Parameter A is defined as the frequency of bioactive fragment occurrence in a protein sequence [28,88,116].

A = a/N,(1)

a—the number of fragments with a given activity in a protein sequence,
N—the number of amino acid residues in a protein.

In the case of the selected activities (ImmD, ImmS, AntiB, AntiF, AntiV, AntiC), the A parameters were computed following the procedures described in Section “Profiles of Potential Bioactivity (ImmD, ImmS, AntiB, AntiF, AntiV, AntiC) of the Analyzed Plant-Derived Proteins”, but using “Calculation” instead of “Profiles of potential biological activity”.

#### 4.2.2. Characterization of Amino Acid Composition of Peptides Exhibiting ImmD or ImmS Activities

The amino acid sequences of the peptides exhibiting ImmD or ImmS activities, deposited and available in the “Bioactive peptides” database (accessed on 1 October 2023), enabled the computation of the number and percentage contents of amino acids occurring in their structures. The amino acid composition was determined using the ProtParam tool (https://web.expasy.org/protparam/) (accessed on August/September 2022 and August/September 2023) [89,90] and also sequences of ImmD or ImmS peptides which were exported from the BIOPEP-UWM database repository (“Bioactive peptides” database) [28,88] (Figure 11).

#### 4.2.3. In Silico Hydrolysis of Plant-Derived Proteins

Simulated and monocatalytic hydrolysis of proteins was performed using the primary structures (amino acid sequences) of the analyzed proteins and specificity of action of the following proteolytic enzymes: ficin (EC 3.4.22.3), stem bromelain (EC 3.4.22.32), or pepsin (pH > 2) (EC 3.4.23.1), as well as tools available in the BIOPEP-UWM database [28,88].

Proteolysis was simulated using the “Enzyme(s) action” app, and the specificity of action of proteolytic enzymes (with one enzyme selected for hydrolysis) was also made available in this app through the BIOPEP-UWM database. This bioinformatic tool became available after opening the “Analysis” panel (“Analysis”—made available for a BIOPEP-UWM database user already after entering the “Proteins” or “Bioactive peptides” database). Next, a protein and an enzyme were selected from the respective lists of these compounds after opening the following windows: “Protein id” and “Enzyme id” (only the enzymes with identification numbers (IDs) of 25, 30, or 39 were selected) and accepting the choice of data for analysis by ticking “View the report with the results”. The resulting “Report of enzyme action” presented hydrolyzed peptide bonds and their location in a protein sequence [28,88].

##### Characterization of Biopeptides (Exhibiting ImmD, ImmS, AntiB, AntiF, AntiV, and AntiC activities) Obtained via Protein Enzymatic Hydrolysates upon Simulated Proteolysis

The “Search for active fragments” app was applied to check if any bioactive peptides were released among the plant protein hydrolysates and to collect information/data about their biological activity. This app became available once the proteolysis simulation had been completed and the “Report of enzyme action” had been generated (Section 4.2.3). The above report provided data about all the released peptides, including detailed information about the identification number (ID) of the biopeptide (deposited in the “Bioactive peptdes” database), sequence, location, name, function, activity, and even monoisotopic mass and chemical mass of the peptide. It further served to derive data related to the peptides exhibiting the six analyzed activities, i.e., ImmD, ImmS, AntiB, AntiF, AntiV, and AntiC [88].

##### Evaluation of the Biological Activity of Proteolysis and Products Obtained upon Simulated Hydrolysis of Plant-Derived Proteins

In order to determine the quantitative parameters characterizing the proteolysis, successive reports were generated by opening the “Calculate A_E_, DH_t_, W, B_E_, V” tab (instead of “Search for active fragments”). This allowed us to compute the theoretical degree of hydrolysis (DH_t_) [28,88,117] and also the quantitative parameters characterizing hydrolysis efficiency, i.e., the frequency of the release of fragments with a given activity by the selected enzymes (parameter A_E_) and the relative frequency of the release of fragments with a given activity by the selected enzymes (parameter W) [28,88,118].

The theoretical degree of hydrolysis (DH_t_) was calculated as follows:

DHt = d/D × 100%,(2)

where d is the number of hydrolyzed peptide bonds in a protein chain and D is the total number of peptide bonds in a protein chain.

The frequency of release of fragments with a given activity by a selected enzyme (parameter A_E_) was calculated as follows:

A_E_ = d/N, (3)

where d is the number of peptides with a given activity (ImmD, ImmS, AntiB, AntiF, AntiV, or AntiC) released by a given enzyme (e.g., ficin) and N is the number of amino acid residues in a protein.

The relative frequency of release of fragments with a given activity by a selected enzyme (parameter W) was calculated as follows:

W = A_E_/A, (4)

where A_E_ is the frequency of release of fragments with a given activity (ImmD, ImmS, AntiB, AntiF, AntiV, or AntiC) by a selected enzyme (from Equation (3)) and A is the frequency of bioactive fragment (ImmD, ImmS, AntiB, AntiF, AntiV, or AntiC) occurrence in a protein sequence (from Equation (1)).

## 5. Conclusions

The activity of the human immune system may be modulated not only by drugs but also by phytocompounds found in plant materials and their proteins, being sources of potential biopeptides exhibiting immunomodulating (ImmD), immunostimulating (ImmS), antimicrobial (antibacterial (AntiB), antifungal (AntiF), and antiviral (AntiV)) activities. Proteins of wheat, rice, buckwheat, and garden peas contained fragments of sequences with ImmD, ImmS, antimicrobial (AntiB, AntiV), and AntiC activities, but none of the proteins possessed fragments with AntiF activity. Among the analyzed proteins of cereals and legumes, the best precursors of immunopeptides exhibiting ImmD activity (YG, YGG, GLF, TPRK) turned out to be those of rice (mean value A = 0.0108) and garden peas (mean value A = 0.0078), whereas the best precursors of ImmS peptides turned out to be buckwheat (GVM, GFL, EAE) (mean value A = 0.0059) and broad bean (LLY, EAE) (mean value A = 0.0053) proteins. Fragments of LLEY and ERF sequences with AntiV activity were identified in garden pea proteins, but the three enzymes used for the simulation of peptide bond hydrolysis did not release peptides exhibiting this activity.

The highest number of immunopeptides, constituted by tyrosine (Y) and glycine (G) and referred to as alpha-lactokinins, was released by stem bromelain upon the simulated hydrolysis of rice proteins (A_E_ = 0.0010–0.0820, W = 0.1994–1.0000, DH_t_ range: 45–82%). Such dipeptides (YG) were also identified in bromelain hydrolysates of wheat, barley, maize, garden pea, peanut, soybean, mouse-ear cress, and cocoa proteins. However, antibacterial peptides (IAK) were released by ficin only from monomers of rice, oat, and garden pea proteins (the DH_t_ range for these proteins was 41–46%). The biopeptides (YG, IAK) identified in protein hydrolysates are potential immunomodulators, nutraceuticals, and components of functional food that may modulate the activity of the human immune system. In turn, stem bromelain and ficin are also active compounds that are primed to release peptide immunomodulators from plant-derived food proteins.

The study results obtained under in silico conditions and presented in this manuscript may also be exploited by the scientific community in order to, e.g., compare our study results with those achieved in future investigations conducted not only in silico but also in vitro, ex vivo, or in vivo conditions and within the scope of the analyzed biological activity/activities, especially considering that certain biopeptides identified using the programs available in the BIOPEP-UWM database were found to be multi-active.

## Figures and Tables

**Table 1:** The content of amino acids (A, R, N, D, C, Q, E, G, H, I) in the sequences of the immunomodulating (ImmD) peptides (from the “Bioactive peptides” database, the BIOPEP-UWM database repository).

Immunomodulating Peptides (ID *)	NBPs ** (NAAR *** Range)	Ala (A)	Arg (R)	Asn (N)	Asp (D)	Cys (C)	Gln (Q)	Glu (E)	Gly (G)	His (H)	Ile (I)
		(Range ****, % *****)^ID^ *									
2575, 2840, **2882**, 2934–2939, 3027, 3056, 3057, 3060, 3083, 3095, **3119**, **3252**, **3469**, **3470**, 3562, 3584, 3608, 3616, 3618, 3620–3626, 3628, 3653–3659, 3662, 3691–3693, 3699–3703, 3734, 3735, 3739, **3741**, 3783, 3793, **3812**, 3864, **8172**, **8176**, 8177, **8498**, **9281**, **9386**, 10030–10034, **10153**, 10370	69 (2–10)	1 (11.1–20.0) ^ID 3060, 3470, 3608, 8498, 10032, 10033^	1–2 (10.0–50.0) ^ID 2575, 2840, 2936, 3027, 3056, 3057, 3083, 3095, 3252, 3562, 3584, 3608, 3616, 3620, 3621, 3624–3626, 3628, 3654, 3655, 3699, 3700, 3701, 3734, 3735, 3739, 3783, 3793, 8177, 10370^	1 (11.1–20.0) ^ID 3119, 3562, 3699, 3701, 3864, 10030–10033^	1–2 (11.1–40.0) ^ID 2938, 2939, 3083, 3616, 3653, 3654, 3656, 3658, 3659, 3699–3701, 3703, 3735, 3812^	1 (16.7) ^ID 2934, 3783^	1–2 (11.1–25.0) ^ID 2943, 3252, 3657, 3658, 3659, 3699–3701, 3735, 3783, 3793, 10030–10033^	1 (11.1–20.0) ^ID 3095, 3470, 3654, 3659, 3662, 3692, 3693, 3701, 8176^	1–2 (10.0–66.7) ^ID 2840, 2882, 2937, 3056, 3057, 3119, 3252, 3469, 3584, 3616, 3620, 3662, 3741, 8498, 9281, 9386, 10033, 10153, 10370^	1 (16.7) ^ID 3783^	1–2 (12.5–33.3) ^ID 2936, 3060, 3119, 3469, 3470, 3618, 3623, 3655, 3658, 3659, 3662, 3692, 3693, 9281, 10370^
Name of peptide (ID *): Tuftsin (2575), oryzatensin (2840), **ImmS peptides (2882, 9386), **ImmD peptides (2934–2939, 3056, 3057, 3060, **3741, 3812, 8498, **10030–10034, 10370), kentsin (3027), splenopentin (3095),**fragment (fr.) of bovine beta-casein (CN) (3119), beta-casokinin-10 (3252), analogue of human beta-CN (3469, 3470), peptide derived from bovine alphaS1-CN fr. 194–199 (8172), peptide derived from ovine colostral whey (8176),**Tuftsin (human Fc region of IgG) (8177), **beta-casomorphin-7 (9281), beta-casomorphin-5 (10153)**											
Including biopeptides from milk proteins:											
**2882, 3119,** **3252, 3469**, **3470, 3741, 3812, 8172, 8176**, **8498**, **9281, 9386, 10153**	**13** **(2–10)**	**1 ** **(16.7)** **^ID 3470, 8498^**	**1 ** **(10.0)** **^ID 3252^**	**1 ** **(16.7)** **^ID 3119^**	**1 ** **(20.0)** **^ID 3812^**	**-**	**2 ** **(20.0)** **^ID 3252^**	**1 ** **(11.1–16.7)** **^ID 3470, 8176 ^**	**1–2 ** **(10.0–66.7)** **^ID 2882, 3119, 3252, 3741, 3469, 8498, 9281, 9386, 10153^**	**-**	**1 ** **(14.3–16.7)** **^ID 3119, 3469, 3470, 9281 ^**
**3059**, 3223, **3228**, 3660, **8174**, 10366–10369, 10371–10373	12 (11–19)	1–4 (6.7–25.0) ^ID 3228, 10366–10369, 10371–10373^	1–5 (5.3–45.5) ^ID 3059, 3223, 8174, 10366, 10367, 10368, 10371, 10373^	1–2 (7.7–11.8) ^ID 3660, 10366, 10371, 10372^	1 (5.9–8.3) ^ID 10366, 10371, 10373^	-	1–2 (5.3–18.2) ^ID 3059, 3223, 3228, 8174, 10367, 10368, 10371^	1 (5.3–15.4) ^ID 3228, 3660, 8174^	1–3 (5.9–17.6) ^ID 3059, 3228, 3660, 8174, 10367, 10369, 10371–10373^	1 (5.9–7.7) ^ID 3228, 10368, 10372^	1–3 (6.2–25.0) ^ID 3059, 3223, 3228, 3660, 8174, 10367, 10371–10373^
Name of peptide (ID *): **ImmS peptide (3059),** C-terminal fragment (fr.) of human receptor of interleukin (3223), **bovine lactoferrin fr. 79–93 (3228), peptide derived from bovine beta-casein (CN) (8174),** ImmD peptides (10366–10369, 10371–10373)											
Including biopeptides from milk proteins:											
**3059,****3228**, **8174**	**3 ** **(15–19)**	**1 ** **(6.7)** **^ID 3228^**	**1 ** **(5.3–5.9)** **^ID 3059, 8174^**	**-**	**-**	**-**	**1–2 ** **(5.3–11.8)** **^ID 3059, 3228, 8174^**	**1 ** **(5.3–6.7)** **^ID 3228, 8174^**	**2 ** **(10.5–13.3)** **^ID 3059, 3228, 8174^**	**1 ** **(6.7)** **^ID 3228^**	**1–2 ** **(6.7–11.8)** **^ID 3059, 3228, 8174^**
**8170**, **8171**, **8173**, **8175**, 10124	5 (>20)	1–2 (3.1–8.0) ^ID 8170, 8175^	2–5 (4.7–20.0) ^ID 8171, 8173, 8175, 10124^	1–3 (2.3–9.4) ^ID 8170, 8171, 8173, 10124^	10 (23.3) ^ID 10124^	2 (4.7–8.0) ^ID 8175, 10124^	1–6 (4.0–14.0) ^ID 8170, 8171, 8175, 10124^	2–7 (4.7–25.0) ^ID 8170, 8171, 8173, 10124^	1–3 (3.6–7.0) ^ID 8171, 8173, 8175, 10124^	1–2 (3.1–8.7) ^ID 8170, 8171, 10124^	1–3 (4.0–10.7) ^ID 8170, 8171, 8173, 8175, 10124^
Name of peptide (ID *): **Peptide derived from bovine alphaS2-casein (CN) (8170), isracidin—peptide derived from alphaS1-CN, fr. 1–23 (8171), peptide derived from bovine beta-CN (8173), bovine lactoferricin B (8175), **lunasin (10124)											
Including biopeptides from milk proteins:											
**8170, 8171, 8173, 8175 **	**4 ** **(23–32)**	**1–2 ** **(3.1–8.0)** **^ID 8170, 8175^**	**2–5 ** **(7.1–20.0)** **^ID 8171, 8173, 8175^**	**2–3 ** **(7.1–9.4)** **^ID 8170, 8171, 8173^**	**-**	**2 ** **(8.0)** **^ID 8175^**	**1–2 ** **(4.0–8.7)** **^ID 8170, 8171, 8175^**	**2–7 ** **(8.7–25.0)** **^ID 8170, 8171, 8173^**	**1 ** **(3.6–4.3)** **^ID 8171, 8173, 8175^**	**1–2 ** **(3.1–8.7)** **^ID 8170, 8171^**	**1–3 ** **(4.0–10.7)** **^ID 8170, 8171, 8173, 8175^**

* ID—identification number in the BIOPEP-UWM database (the “Bioactive peptides” database), ** NBPs—the number of biopeptides (BPs) (accessed on August/September 2022 and August/September 2023; the data were updated as of 1 October 2023), *** NAAR—the number of amino acid residues found in the analyzed BPs. **** Range, ***** (%)—amino acid sequences of the peptides exhibiting ImmD activities, deposited and available in the “Bioactive peptides” database (accessed on 1 October 2023), enabling computation of the number and percentage content of amino acids occurring in their structures. The amino acid composition was determined using the ProtParam tool (https://web.expasy.org/protparam/, (accessed on August/September 2022 and August/September 2023. The data were updated as of 1 October 2023)) and also sequences of ImmD peptides which were exported from the BIOPEP-UWM database repository (“Bioactive peptides” database). Amino acids presented in the form of a three- and single-letter amino acid code: Ala (A)—alanine, Arg (R)—arginine, Asn (N)—asparagine, Asp (D)—aspartic acid, Cys (C)—cysteine, Gln (Q)—glutamine, Glu (E)—glutamic acid, Gly (G)—glycine, His (H)—histidine, Ile (I)—isoleucine. The bold font in the **blue**color denotes biopeptides derived from **milk proteins**.

**Table 2:** The content of amino acids (L, K, M, F, P, S, T, W, Y, V) in the sequences of the immunomodulating (ImmD) peptides (from the “Bioactive peptides” database, the BIOPEP-UWM database repository).

Immunomodulating Peptides (ID *)	NBPs ** (NAAR *** Range)	Leu (L)	Lys (K)	Met (M)	Phe (F)	Pro (P)	Ser (S)	Thr (T)	Trp (W)	Tyr (Y)	Val (V)
		(Range ****, % *****)^ID^ *									
2575, 2840, **2882, **2934–2939, 3027, 3056, 3057, 3060, 3083, 3095, **3119**, **3252**, **3469, 3470****,** 3562, 3584, 3608, 3616, 3618, 3620–3626, 3628, 3653–3659, 3662, 3691–3693, 3699–3703, 3734, 3735, 3739, **3741, **3783, 3793, **3812,** 3864, **8172, 8176**, 8177, **8498**, **9281**, **9386, **10030–10034, **10153, **10370	69 (2–10)	1–2 (10.0–33.3) ^ID 2840, 2934, 2935, 2937, 3056, 3057, 3252, 3616, 3653, 3654, 3659, 8172, 8176, 9386, 10030–10034^	1–3 (14.3–50.0) ^ID 2575, 3027, 3056, 3057, 3060, 3095, 3562, 3584, 3608, 3616, 3618, 3620, 3621–3626, 3628, 3653, 3657, 3691–3693, 3699–3702, 3734, 3735, 3739, 3812, 3864, 8177^	1 (11.1–16.7) ^ID 2840, 2936, 8172^	1–2 (11.1–40.0) **^ID^** ^2936, 2937, 3656–3659, 3691, 3701–3703, 3812, 8176, 9281, 9386, 10030, 10031, 10033, 10034, 10153^	1–3 (16.7–50.0) ^ID 2575, 2840, 2934–2939, 3027, 3083, 3119, 3252, 3469, 3470, 3618, 3620, 3621–3626, 3628, 3653, 3655, 3692, 3693, 3739, 3783, 3793, 3864, 8172, 8176, 8177, 9281, 10030–10034, 10153, 10370^	1–2 (11.1–40.0) ^ID 3060, 3083, 3584, 3662, 3734, 3812, 8176, 10032^	1–2 (12.5–33.3) ^ID 2575, 2936, 2937, 3027, 3608, 3618, 3622–3624, 3653, 3654, 3656–3659, 3662, 3691–3693, 3702, 3703, 3739, 8172, 8498, 10032, 10034^	1 (16.7) ^ID 2935, 8172, 8498^	1–2 (10.0–50.0) ^ID 2840, 2882, 2934, 2935, 2939, 3057, 3095, 3252, 3469, 3470, 3562, 3656, 3657, 3691, 3702, 3703, 3741, 3864, 8176, 8177, 8498, 9281, 10153, 10370^	1–2 (11.1–40.0) ^ID 2934, 2935, 2938, 2939, 3056, 3057, 3060, 3095, 3252, 3469, 3562, 3608, 3628, 3656, 3657, 3691, 3702, 3703, 8176, 8498, 10034^
Name of peptide (ID *): Tuftsin (2575), oryzatensin (2840), **ImmS peptides (2882, 9386), **ImmD peptides (2934–2939, 3056, 3057, 3060, **3741, 3812, 8498, **10030–10034, 10370), kentsin (3027), splenopentin (3095),**fragment (fr.) of bovine beta-casein (CN) (3119), beta-casokinin-10 (3252), analogue of human beta-CN (3469, 3470), peptide derived from bovine alphaS1-CN, fr. 194–199 (8172), peptide derived from ovine colostral whey (8176),**Tuftsin (human Fc region of IgG) (8177), **beta-casomorphin-7 (9281), beta-casomorphin-5 (10153)**											
Including biopeptides from milk proteins:											
**2882, 3119,** **3252, 3469**, **3470, 3741, 3812, 8172, 8176**, **8498**, **9281, 9386, 10153**	**13** **(2–10)**	**1 ** **(10.0–33.3)** **^ID 3252, 8172, 8176, 9386^**	**1 ** **(20.0)** **^ID 3812^**	**1 ** **(16.7)** **^ID 8172^**	**1–2 ** **(11.1–40.0)** **^ID 3812, 8176, 9281, 9386, 10153^**	**1–3 ** **(16.7–50.0)** **^ID 3119, 3252, 3469, 3470, 8172, 8176, 9281, 10153^**	**1 ** **(11.1–20.0)** **^ID 3812, 8176^**	**1–2 ** **(16.7–33.3)** **^ID 8172, 8498^**	**1 ** **(16.7)** **^ID 8172, 8498^**	**1 ** **(10.0–50.0)** **^ID 2882, 3252, 3469, 3470, 3741, 8176, 8498, 9281, 10153^**	**1–2 ** **(16.7–22.2)** **^ID 3252, 3469, 8176, 8498 ^**
**3059**, 3223, **3228**, 3660, **8174**, 10366–10369, 10371–10373	12 (11–19)	1–4 (5.9–30.8) ^ID 3059, 3660, 8174, 10366–10369^	1–2 (5.9–11.8) ^ID 3223, 3228, 3660, 10369, 10371–10373^	1 (5.9–7.7) ^ID 10369, 10372, 10373^	1–2 (5.3–12.5) ^ID 3059, 8174, 10366–10369, 10372^	1–4 (5.9–23.5) ^ID 3059, 3228, 3660, 8174, 10366–10369, 10371, 10372^	1–3 (5.9–23.1) ^ID 3223, 3228, 3660, 10366–10369, 10372, 10373^	1–3 (5.9–23.1) ^ID 3223, 3228, 3660, 10369, 10371, 10372^	-	1–3 (5.3–20.0) ^ID 3059, 3228, 8174, 10366, 10373^	1–4 (5.9–23.5) ^ID 3059, 8174, 10366, 10372, 10373^
Name of peptide (ID *): **ImmS peptide (3059),** C-terminal fragment (fr.) of human receptor of interleukin (3223), **bovine lactoferrin fr. 79–93 (3228), peptide derived from bovine beta-casein (CN) (8174)**, ImmD peptides (10366–10369, 10371–10373)											
Including biopeptides from milk proteins:											
**3059,****3228**, **8174**	**3** **(15–19)**	**1–3 ** **(5.9–15.8)** **^ID 3059, 8174^**	**1 ** **(6.7)** **^ID 3228^**	**-**	**1 ** **(5.3–5.9)** **^ID 3059, 8174^**	**1–4 ** **(6.7–23.5)** **^ID 3059, 3228, 8174^**	**1 ** **(6.7)** **^ID 3228^**	**2** **(13.3)** **^ID 3228^**	**-**	**1–3 ** **(5.3–20.0)** **^ID 3059, 3228, 8174^**	**3 ** **(15.8–17.6)** **^ID 3059, 8174^**
**8170**, **8171**, **8173**, **8175**, 10124	5 (>20)	1–4 (4.0–17.4) ^ID 8171, 8173, 8175, 10124^	1–4 (3.6–12.5) ^ID 8170, 8171, 8173, 8175, 10124^	1–2 (2.3–6.2) ^ID 8170, 8175, 10124^	1–2 (4.3–8.0) ^ID 8171, 8175^	1–3 (2.3–13.0) ^ID 8170, 8171, 8173, 8175, 10124^	1–6 (4.0–18.8) ^ID 8170, 8173, 8175, 10124^	1–2 (2.3–6.2) ^ID 8170, 8173, 8175, 10124^	1–2 (2.3–8.0) ^ID 8175, 10124^	1 (3.1) ^ID 8170^	1–2 (3.1–16.7) ^ID 8170, 8171, 8173, 8175, 10124^
Name of peptide (ID*): **Peptide derived from bovine alphaS2-casein (CN) (8170), Isracidin—peptide derived from alphaS1-CN, fragment 1–23 (8171), peptide derived from bovine beta-CN (8173), bovine lactoferricin B** (**8175**), lunasin (10124)											
Including biopeptides from milk proteins:											
**8170, 8171, 8173, 8175**	**4 ** **(23–32)**	**1–4 ** **(4.0–17.4)** **^ID 8171, 8173, 8175^**	**1–4 ** **(3.6–12.5)** **^ID 8170, 8171, 8173, 8175^**	**1–2 ** **(4.0–6.2)** **^ID 8170, 8175^**	**1–2 ** **(4.3–8.0)** **^ID 8171, 8175^**	**1–3 ** **(3.1–13.0)** **^ID^ ^8170, 8171, 8173, 8175^ **	**1–6 ** **(4.0–18.8)** **^ID 8170, 8173, 8175^**	**1–2 ** **(3.6–6.2)** **^ID 8170, 8173, 8175^**	**2 ** **(8.0)** **^ID 8175^**	**1 ** **(3.1)** **^ID 8170^**	**1–2 ** **(3.1–7.1)** **^ID 8170, 8171, 8173, 8175^**

* ID-identification number in the BIOPEP-UWM database (the “Bioactive peptides” database), ** NBPs—the number of biopeptides (BPs) (accessed on August/September 2022 and August/September 2023; the data were updated as of 1 October 2023), *** NAAR—the number of amino acid residues found in the analyzed BPs. **** Range, ***** (%)—the amino acid sequences of the peptides exhibiting ImmD activities, deposited and available in the “Bioactive peptides” database (accessed on 1 October 2023), enabling computation of the number and percentage contents of amino acids occurring in their structures. The amino acid composition was determined using the ProtParam tool (https://web.expasy.org/protparam/, (accessed on August/September 2022 and August/September 2023. The data were updated as of 1 October 2023)) and also sequences of ImmD peptides, which were exported from the BIOPEP-UWM database repository (“Bioactive peptides” database). Amino acids presented in the form of a three- and single-letter amino acid code: Leu (L)—leucine, Lys (K)—lysine, Met (M)—methionine, Phe (F)—phenylalanine, Pro (P)—proline, Ser (S)—serine, Thr (T)—threonine, Trp (W)—tryptophan, Tyr (Y)—tyrosine, Val (V)—valine. The bold font in the **blue**color denotes biopeptides derived from **milk proteins**.

**Table 3:** The content of amino acids (A, R, N, D, C, Q, E, G, H, I) in the sequences of the immunostimulating (ImmS) peptides (from the “Bioactive peptides” database, the BIOPEP-UWM database repository).

Immunostimulating Peptides (ID *)	NBPs ** (NAAR *** Range)	Ala (A)	Arg (R)	Asn (N)	Asp (D)	Cys (C)	Gln (Q)	Glu (E)	Gly (G)	His (H)	Ile (I)
		(Range ****, % *****)^ID^ *									
3054, **3061, 3064**, **3065**, 3066, **3098, 3099,** 3120, 3613–3615, 3708, 3710, 3984, 3985	15 (3–9)	1 (20.0–33.3) ^ID 3066, 3120^	1–2 (11.1–40.0) ^ID 3054, 3613–3615, 3708, 3710, 3984, 3985^	1 (11.1–20.0) ^ID 3615, 3708, 3710, 3984, 3985^	1 (20.0) ^ID 3054, 3613^	-	-	1–3 (16.7–66.7) ^ID 3064, 3066, 3120^	1 (12.5–33.3) ^ID 3061, 3098, 3099, 3613, 3984, 3985^	-	1 (12.5–16.7) ^ID 3064, 3984, 3985^
Name of peptide (ID *): Thymopentin (3054), **ImmS peptides (3061, 3064, 3099), fragment of bovine beta-casein (CN) (3065, 3098)**, ImmS peptide (3066), alpha-thymosin-1 K23 (3120)											
Including biopeptides from milk proteins:											
**3061, 3064, 3065, 3098, 3099**	**5** **(3–6)**	**-**	**-**	**-**	**-**	**-**	**-**	**1 ** **(16.7)** **^ID 3064^**	**1 ** **(33.3)** **^ID 3061, 3098, 3099^**	**-**	**1 ** **(16.7)** **^ID 3064^**
3709, 3711	2 (13)	-	2 (15.4) ^ID 3709, 3711^	1 (7.7) ^ID 3709, 3711^	1 (7.7) ^ID 3709, 3711^	-	-	-	-	-	-

* ID—identification number in the BIOPEP-UWM database (the “Bioactive peptides” database), ** NBPs—the number of biopeptides (BPs) (accessed on August/September 2022 and August/September 2023; the data were updated as of 1 October 2023), *** NAAR—the number of amino acid residues found in the analyzed BPs. **** Range, ***** (%)—the amino acid sequences of the peptides exhibiting ImmS activities, deposited and available in the “Bioactive peptides” database (accessed on 1 October 2023), enabling computation of the number and percentage content of amino acids occurring in their structures. The amino acid composition was determined using the ProtParam tool (https://web.expasy.org/protparam/, (accessed on August/September 2022 and August/September 2023. The data were updated as of 1 October 2023)) and also sequences of ImmD peptides which were exported from the BIOPEP-UWM database repository (“Bioactive peptides” database). Amino acids presented in the form of a three- and single-letter amino acid code: Ala (A)—alanine, Arg (R)—arginine, Asn (N)—asparagine, Asp (D)—aspartic acid, Cys (C)—cysteine, Gln (Q)—glutamine, Glu (E)—glutamic acid, Gly (G)—glycine, His (H)—histidine, Ile (I)—isoleucine. The bold font in the **blue** color denotes biopeptides derived from **milk proteins**.

**Table 4:** The content of amino acids (L, K, M, F, P, S, T, W, Y, V) in the sequences of the immunostimulating (ImmS) peptides (from the “Bioactive peptides” database, the BIOPEP-UWM database repository).

Immunostimulating Peptides (ID *)	NBPs ** (NAAR *** Range)	Leu (L)	Lys (K)	Met (M)	Phe (F)	Pro (P)	Ser (S)	Thr (T)	Trp (W)	Tyr (Y)	Val (V)
		(Range ****, % *****)^ID^ *									
3054, **3061, 3064**, **3065**, 3066, **3098, 3099,** 3120, 3613–3615, 3708, 3710, 3984, 3985	15 (3–9)	1–2 (11.1–66.7) ^ID 3061, 3065, 3099, 3613, 3708, 3710, 3984, 3985^	1–2 (20.0–22.2) ^ID 3054, 3120, 3613, 3614, 3708, 3710^	1 (11.1–33.3) ^ID 3098, 3710, 3984, 3985^	1–2 (22.2–33.3) ^ID 3061, 3708, 3710^	1–2 (11.1–33.3) ^ID 3064, 3614, 3708, 3710, 3984, 3985^	1 (12.5) ^ID 3984^	1 (20.0) ^ID 3614^	-	1 (16.7–33.3) ^ID 3054, 3064, 3065,^ ^3099^	1–2 (11.1–40.0) ^ID 3054, 3064, 3098, 3614, 3615, 3708^
Name of peptide (ID *): Thymopentin (3054), **ImmS peptides (3061, 3064, 3099), fragment of bovine beta-casein (CN) (3065, 3098**), ImmS peptide (3066), alpha-thymosin-1 K23 (3120)											
Including biopeptides from milk proteins:											
**3061, 3064, 3065, 3098, 3099**	**5** **(3–6)**	**1–2 ** **(33.3–66.7)** **^ID 3061, 3065, 3099^**	**-**	**1 ** **(33.3)** **^ID 3098^**	**1 ** **(33.3)** **^ID 3061^**	**2 ** **(33.3)** **^ID 3064^**	**-**	**-**	**-**	**1 ** **(16.7–33.3)** **^ID 3064, 3065, 3099^**	**1 ** **(16.7–33.3)** **^ID 3064, 3098^**
3709, 3711	2 (13)	1 (7.7) ^ID 3709, 3711^	2 (15.4) ^ID 3709, 3711^	1 (7.7) ^ID 3709^	2 (15.4) ^ID 3709, 3711^	1 (7.7) ^ID 3709, 3711^	2 (15.4) ^ID 3709, 3711^	-	-	-	1 (7.7) ^ID 3711^

* ID—identification number in the BIOPEP-UWM database (the “Bioactive peptides” database), ** NBPs—the number of biopeptides (BPs) (accessed on August/September 2022 and August/September 2023; the data were updated as of 1 October 2023), *** NAAR—the number of amino acid residues found in the analyzed BPs. **** Range, ***** (%)—the amino acid sequences of the peptides exhibiting ImmS activities, deposited and available in the “Bioactive peptides” database (accessed on 1 October 2023), enabling computation of the number and percentage content of amino acids occurring in their structures. The amino acid composition was determined using the ProtParam tool (https://web.expasy.org/protparam/, (accessed on August/September 2022 and August/September 2023. The data were updated as of 1 October 2023)) and also sequences of ImmS peptides, which were exported from the BIOPEP-UWM database repository (“Bioactive peptides” database). Amino acids presented in the form of three- and single-letter amino acid codes: Leu (L)—leucine, Lys (K)—lysine, Met (M)—methionine, Phe (F)—phenylalanine, Pro (P)—proline, Ser (S)—serine, Thr (T)—threonine, Trp (W)—tryptophan, Tyr (Y)—tyrosine, Val (V)—valine. The bold font in the **blue**color denotes biopeptides derived from **milk proteins**.

**Table 5:** Bioparameters characterizing the in silico hydrolysis of ^1^ wheat, ^2^ barley, ^3^ oat, and ^4^ maize proteins and sequences of biopeptides released by stem bromelain and ficin *.

Protein Groups (NPs **)^ID^ ***	ImmD **** /AntiB * Peptides (seq)^ID^ ***	(Bioparameters)^ID^ ***		
		DH_t_ (%) *****	A_E_ ******	W *******
^1 ^Alpha-gliadin (1)^ID 1420^/ ^1 ^Alpha/beta-gliadin (1)^ID 1179^/ ^1 ^Glutenin, LMW-subunits (2)^ID 1394, 1395^	(YG)^ID 1420^/ (YG)^ID 1179^/ (YG)^ID 1394, 1395^	(29.07)^ID 1420^/ (32.35)^ID 1179^/ (37.13)^ID 1394^ (36.54)^ID 1395^	(0.0034)^ID 1420^/ (0.0033)^ID 1179^/ (0.0029)^ID 1394^ (0.0027)^ID 1395^	(1.0000)^ID 1420^/ (1.0000)^ID 1179^/ (1.0000)^ID 1394, 1395^
^2 ^ Hordeins (2) ^ID 1633, 1645^	(YG)^ID 1633, 1645^	(53.41)^ID 1633^ (61.38)^ID 1645^	(0.0020)^ID 1633^ (0.0068)^ID 1645^	(1.0000)^ID 1633, 1645^
^3 ^ Avenin (1)^ID 1452^	(IAK *)^ID 1452^	(45.07 *)^ID 1452^	(0.0047 *)^ID 1452^	(1.0000 *)^ID 1452^
^4 ^ Legumin 1 (1) ^ID 1156^	(3xYG)^ID 1156^	(57.26)^ID 1156^	(0.0062)^ID 1156^	(0.5962)^ID 1156^

*—the biopeptides released by ficin, ** NPs—the number of proteins with bioactive sequences (biopeptides) released by the enzyme, *** ID—identification number in the BIOPEP-UWM database, **** ImmD/AntiB peptides (seq)—immunomodulatory/antibacterial peptides (sequence of biopeptides), amino acid sequences presented in the form of a single-letter amino acid code: A—alanine, G—glycine, I—isoleucine, K—lysine, Y—tyrosine), ***** DH_t_—the theoretical degree of hydrolysis (%), ****** A_E_—the parameter A_E_ (the frequency of release of fragments with a given activity by a selected enzyme), ******* W—the parameter W (the relative frequency of release of fragments with a given activity by a selected enzyme, defined in the “Materials and Methods” section). LMW—low molecular weight.

**Table 6:** Bioparameters characterizing the in silico hydrolysis of rice proteins and sequences of biopeptides released by stem bromelain and ficin *.

Protein Groups (NPs **)^ID^ ***	ImmD **** /AntiB * Peptides (seq)^ID^ ***	(Bioparameters)^ID^ ***		
		DH_t_ (%) *****	A_E_ ******	W *******
Prolamins (4) ^ID 1152, 1154, 1155, 1581^	(YG)^ID 1152, 1154, 1155, 1581^	(53.38)^ID 1152^ (54.42)^ID 1154^ (54.36)^ID 1155^ (45.00)^ID 1581^	(0.0067)^ID 1152, 1155^ (0.0068)^ID 1154^ (0.0099)^ID 1581^	(0.1994)^ID 1152^ (0.2012)^ID 1154, 1155^ (1.0000)^ID 1581^
Glutelins (3) ^ID 1537, 1539, 1540^	(YG)^ID 1537, 1539, 1540^	(52.01)^ID 1537, 1539^ (52.12)^ID 1540^	(0.0020)^ID 1537, 1539, 1540^	(0.5000)^ID 1537, 1539, 1540^
Oryzains+expansin-B1 (4) ^ID 1547, 1548, 1549, 1772^	(YG)^ID 1547, 1772^ (2xYG)^ID 1548, 1549^	(53.83) ^ID 1547^ (49.89)^ID 1548^ (59.28)^ID 1549^ (47.74)^ID 1772^	(0.0021)^ID 1547^ (0.0044)^ID 1548^ (0.0055)^ID 1549^ (0.0037)^ID 1772^	(1.0000)^ID 1547, 1549^ (0.6667)^ID 1548^ (0.4933)^ID 1772^
Oleosin (1) ^ID 1545^	(YG)^ID 1545^	(64.63)^ID 1545^	(0.0068)^ID 1545^	(1.0000)^ID 1545^
Glycine-rich proteins + other1 (5) ^ID 1541, 1542, 1562, 1563, 1564^	(9xYG)^ID 1541, 1562, 1563^ (15xYG)^ID 1542^ (YG)^ID 1564^	(79.88)^ID 1541^ (82.42)^ID 1542^ (69.57)^ID 1562^ (68.13)^ID 1563^ (67.32)^ID 1564^	(0.0545)^ID 1541^ (0.0820)^ID 1542^ (0.0556)^ID 1562^ (0.0559)^ID 1563^ (0.0065)^ID 1564^	(1.0000)^ID 1541, 1564^ (0.8827)^ID 1542^ (0.5628)^ID 1562^ (0.6425)^ID 1563^
G10 Homolog protein (1) ^ID 1534^	(IAK *)^ID 1534^	(45.77 *)^ID 1534^	(0.0070 *)^ID 1534^	(1.0000 *)^ID 1534^
Enzymes + other2 (7) ^ID 1703, 1713, 1714, 1719, 1721−1723^	(YG)^ID 1703^ (2xYG, IKPR)^ID 1713, 1714^ (YG)^ID 1719, 1721, 1722, 1723^	(59.67)^ID 1703^ (53.49)^ID 1713^ (53.50)^ID 1714^ (56.18)^ID 1719^ (56.14)^ID 1721^ (54.30)^ID 1722^ (55.56)^ID 1723^	(0.0010)^ID 1703, 1719, 1721^ (0.0030)^ID 1713, 1714^ (0.0011)^ID 1722^ (0.0012)^ID 1723^	(0.4762)^ID 1703^ (0.7500)^ID 1713^ (0.7317)^ID 1714^ (0.4762)^ID 1719^ (1.0000)^ID 1721, 1723^ (0.5238)^ID 1722^
Actins + other3 (11) ^ID 1529, 1530, 1531, 1532, 1686, 1693, 1697, 1698, 1708, 1709, 1715^	(YG)^ID 1529, 1530, 1531, 1532, 1686, 1693, 1697, 1698, 1709, 1715^ (2xYG)^ID 1708^	(49.73)^ID 1529^ (50.79)^ID 1530^ (50.13)^ID 1531, 1532^ (61.34)^ID 1686^ (55.30)^ID 1693^ (58.52)^ID 1697, 1698^ (65.76)^ID 1708^ (69.66)^ID 1709^ (64.33)^ID 1715^	(0.0027)^ID 1529, 1531, 1532^ (0.0026)^ID 1530, 1693^ (0.0022)^ID 1686^ (0.0028)^ID 1697, 1698^ (0.0042)^ID 1708^ (0.0031)^ID 1709^ (0.0021)^ID 1715^	(1.0000)^ID 1529, 1530, 1531, 1532, 1693, 1697, 1698, 1708, 1709, 1715^ (0.3385)^ID 1686^
Other4 (1) ^ID 1591^	(YG)^ID 1591^	(51.91)^ID 1591^	(0.0029)^ID 1591^	(0.5000)^ID 1591^

*—the biopeptides released by ficin, ** NPs—the number of proteins with bioactive sequences (biopeptides) released by the enzyme, *** ID—identification number in the BIOPEP-UWM database, **** ImmD/AntiB peptides (seq)—immunomodulatory/antibacterial peptides (sequence of biopeptides), amino acid sequences presented in the form of a single-letter amino acid code: A—alanine, R—arginine, G—glycine, I—isoleucine, K—lysine, P—proline, Y—tyrosine), ***** DH_t_—the theoretical degree of hydrolysis (%), ****** A_E_—the parameter A_E_ (the frequency of release of fragments with a given activity by the selected enzyme), ******* W—the parameter W (the relative frequency of release of fragments with a given activity by the selected enzyme, defined in the “Materials and Methods” section).

**Table 7:** Bioparameters characterizing the in silico hydrolysis of garden pea proteins and sequences of biopeptides released by stem bromelain and ficin *.

Protein Groups (NPs **)^ID^ ***	ImmD **** /AntiB * Peptides (seq)^ID^ ***	(Bioparameters)^ID^ ***		
		DH_t_ (%) *****	A_E_ ******	W *******
Albumin (1)^ID 1490^/ Actins (3)^ID 1481–1483^/ Histones (3)^ID 1497, 1498, 1500^	(2xYG)^ID 1490^/ (YG)^ID 1481–1483^/ (YG)^ID 1497, 1498, 1500^	(49.57)^ID 1490^/ (50.40)^ID 1481^ (50.67)^ID 1482^ (50.80)^ID 1483^/ (62.42)^ID 1497^ (63.51)^ID 1498^ (64.36)^ID 1500^	(0.0087)^ID 1490^/ (0.0027)^ID 1481–1483^/ (0.0067)^ID 1497, 1498^ (0.0098)^ID 1500^	(1.0000)^ID 1490^/ (1.0000)^ID 1481–1483^/ (0.5038)^ID 1497^ (0.3333)^ID 1498^ (0.5000)^ID 1500^
Disease resistance response proteins (3) ^ID 1494–1496^	(IAK *)^ID 1494–1496^	(43.04 *)^ID 1494^ (43.31 *)^ID 1495^ (40.76 *)^ID 1496^	(0.0063 *)^ID 1494–1496^	(1.0000 *)^ID 1494, 1495^ (0.4961 *)^ID 1496^
Germin-like proteins (2)^ID 1526, 1527^/ Other (1)^ID 1522^	(YG)^ID 1526, 1527^/ (2xYG)^ID 1522^	(53.61)^ID 1526^ (52.58)^ID 1527^/ (51.88)^ID 1522^	(0.0051)^ID 1526, 1527^/ (0.0038)^ID 1522^	(1.0000)^ID 1526, 1527^/ (0.4043)^ID 1522^

*—the biopeptides released by ficin, ** NPs–the number of proteins with bioactive sequences (biopeptides) released by the enzyme, *** ID–identification number in the BIOPEP-UWM database, **** ImmD/AntiB peptides (seq)–immunomodulatory/antibacterial peptides (sequence of biopeptides, amino acid sequences presented in the form of a single-letter amino acid code: A—alanine, G—glycine, I—isoleucine, K—lysine, Y—tyrosine), ***** DH_t_—the theoretical degree of hydrolysis (%), ****** A_E_—the parameter A_E_ (the frequency of release of fragments with a given activity by a selected enzyme), ******* W—the parameter W (the relative frequency of release of fragments with a given activity by selected enzyme, defined in the “Materials and Methods” section).

**Table 8:** Bioparameters characterizing the in silico hydrolysis of soybean, peanut, mouse-ear cress, and cocoa proteins and sequences of biopeptides released by stem bromelain and ficin *.

Protein Groups (NPs **)^ID^ ***	ImmD **** /AntiB * Peptides (seq)^ID^***	(Bioparameters)^ID^ ***		
		DH_t_ (%) *****	A_E_ ******	W *******
Soybean (1) ^ID 1776^	(YG)^ID 1776^	(48.94)^ID 1776^	(0.0026)^ID 1776^	(0.4906)^ID 1776^
Peanut (5) ^ID 1781, 1782, 1779, 1785, 1780^	(YG)^ID 1781, 1782, 1779, 1785, 1780^	(50.40)^ID 1781^ (50.28)^ID 1782^ (48.45)^ID 1779^ (50.76)^ID 1785^ (44.44)^ID 1780^	(0.0020)^ID 1781^ (0.0019)^ID 1782, 1785^ (0.0016)^ID 1779^ (0.0058)^ID 1780^	(0.5128)^ID 1781^ (0.5000)^ID 1782, 1785^ (1.0000)^ID 1779, 1780^
Mouse-ear cress (1) ^ID 1140^	(YG)^ID 1140^	(59.44)^ID 1140^	(0.0028)^ID 1140^	(0.3333)^ID 1140^
Cocoa storage protein (1) ^ID 1114^	(YG)^ID 1114^	(51.48)^ID 1114^	(0.0025)^ID 1114^	(0.5102)^ID 1114^

*—the biopeptides released by ficin (lack), ** NPs—the number of proteins with bioactive sequences (biopeptides) released by the enzyme, *** ID—identification number in the BIOPEP-UWM database, **** ImmD/AntiB peptides (seq)–immunomodulatory/antibacterial peptides (sequence of biopeptides), amino acid sequences presented in the form of a single-letter amino acid code: G—glycine, Y—tyrosine), ***** DH_t_—the theoretical degree of hydrolysis (%), ****** A_E_—the parameter A_E_ (the frequency of the release of fragments with a given activity by the selected enzyme), ******* W—the parameter W (the relative frequency of the release of fragments with a given activity by the selected enzyme, defined in the “Materials and Methods” section).

## Data Availability

The protein/peptide sequences were deposited in the BIOPEP-UWM database (https://biochemia.uwm.edu.pl/biopep-uwm/, accessed on August/September 2022 and August/September 2023).

## Appendix A

**Table A1:** Characteristics of the selected activities of wheat (^I^ *Triticum aestivum*, ^II^ *Triticum aestivum* subsp. *Spelta*, ^III^ *Triticum aestivum* spp. *sphaeroccoum*, ^IV^ *Triticum aestivum* spp. *compactum*, ^V^ *Triticum aestivum* spp. *tibeticum*, ^VI^ *Triticum turgidum* subsp. *durum*) proteins.

Protein Groups (NAAR *)^ID^ **	ImmD (A ***, seq ****)^ID^ **	ImmS (A ***, seq ****)^ID^ **	AntiB (A ***, seq ****)^ID^ **	AntiF (A ***, seq ****)^ID^ **	AntiV (A ***, seq ****)^ID^ **	AntiC (A ***, seq ****)^ID^ **
^I,II^ Alpha-gliadins (21^T^) (287)^ID 1304^, (313)^ID 1305^, (288)^ID 1306^, (289)^ID 1307^, (259,f)^ID 1308^, (318,pr)^ID 1310^, (290)^ID 1420^, (269)^ID 1421^, (277)^ID 1422^, (270)^ID 1423^, (276)^ID 1424^, (278)^ID 1425^, (276)^ID 1426^, (265)^ID 1427^, (273)^ID 1428^, (274)^ID 1429^, (265)^ID 1430^, (288,pr,f)^ID 1434^ Proteins ***** (54,f)^ID 1435, 1436^, (24,f)^ID 1431^	(0.0034, YG)^ID 1420^	-	-	-	(0.0038, ERF)^ID 1430^	-
^I^ Alpha/beta-gliadins (12^T^) (262,pr)^ID 1177^, (291,pr)^ID 1178^, (307,pr)^ID 1179^, (282,pr)^ID 1180^, (319,pr)^ID 1181^, (296,pr)^ID 1182^, (297,pr)^ID 1183^, (313,pr)^ID 1184^, (186,f)^ID 1186^, (286,pr)^ID 1278^, (313,pr)^ID 1311^, (120,f)^ID 1365^	(0.0033, YG)^ID 1179^	-	-	-	-	-
^I,III,IV^ Gamma-gliadins (38^T^) (304,pr)^ID 1145^, (327,pr)^ID 1146^, (291,pr)^ID 1147^, (244,pr)^ID 1148^, (291,pr)^ID 1185^, (251,pr,f)^ID 1187^, (279,f)^ID 1309^, (282)^ID 1317^, (257)^ID 1318^, (311)^ID 1319^, (274)^ID 1320^, (275)^ID 1321^, (146,f)^ID 1364^, (279)^ID 1366^, (279)^ID 1367^, (279)^ID 1368^, (279)^ID 1369^, (213,f)^ID 1396^, (327)^ID 1397^, (298)^ID 1398^, (302)^ID 1399^, (285)^ID 1400^, (203,f)^ID 1401^, (337)^ID 1402^, (256)^ID 1403^, (241,f)^ID 1404^, (267,f)^ID 1409^, (244,f)^ID 1410^, (259,f)^ID 1411^, (270,f)^ID 1412^, (283,f)^ID 1413^, (264,f)^ID 1414^, (259,f)^ID 1415^, (250,f)^ID 1416^, (252,f)^ID 1417^, (277)^ID 1419^ Proteins ***** (15,f)^ID 1437, 1438^	-	-	-	-	-	-
^I^ Omega-gliadins (3^T^) (280)^ID 1418^ Proteins ***** (28,f)^ID 1189^, (32,f)^ID 1340^	(0.0036, YG)^ID 1418^	-	-	-	-	-
^I^ Glutenin, HMW-subunits (33^T^) (817)^ID 1110^, (101,f)^ID 1279^, (660,pr)^ID 1281^, (839,pr)^ID 1282^, (655)^ID 1316^, (836)^ID 1322^, (180,f)^ID 1323^, (188,f)^ID 1324^, (188,f)^ID 1325^, (188,f)^ID 1326^, (660)^ID 1327^, (661)^ID 1328^, (161)^ID 1342^, (137,f)^ID 1343^, (137,f)^ID 1344^, (119,f)^ID 1345^, (114,f)^ID 1346^, (184,f)^ID 1347^, (209,f)^ID 1348^, (973)^ID 1353^, (201,f)^ID 1356^, (211,f)^ID 1357^, (220,f)^ID 1358^, (238,f)^ID 1359^, (181,f)^ID 1360^, (213,f)^ID 1361^, (191,f)^ID 1362^, (196,f)^ID 1363^, (720,pr, subunit y)^ID 1405^, (811,pr, subunit x)^ID 1406^, (713,pr, subunit y)^ID 1407^, (462,pr, subunit x)^ID 1408^ Protein ***** (39,f)^ID 1280^	(0.0073, YG)^ID 1343, 1344^	(0.0012, LGY)^ID 1406^	-	-	-	(0.0012, VVV)^ID 1110^, (0.0024, 2xVVV)^ID 1282, 1322^, (0.0111, 2xVVV)^ID 1323^, (0.0106, 2xVVV)^ID 1324, 1325, 1326^, (0.0062, VVV)^ID 1342^, (0.0084, VVV)^ID 1345^, (0.0088, VVV)^ID 1346^, (0.0048, VVV)^ID 1348^, (0.0021, 2xVVV)^ID 1353^, (0.0084, 2xVVV)^ID 1359^, (0.0047, VVV)^ID 1361^, (0.0105, 2xVVV)^ID 1362^, (0.0012, VVV)^ID 1406^, (0.0022, VVV)^ID 1408^
^I,V^ Glutenin, LMW-subunits (57^T^) (356,pr)^ID 1283^, (306,pr)^ID 1284^, (295,pr)^ID 1285^, (285,f)^ID 1297^, (373,f)^ID 1298^, (298)^ID 1299^, (307)^ID 1300^, (303)^ID 1301^, (306)^ID 1302^, (359)^ID 1303^, (229)^ID 1312^, (261)^ID 1313^, (276)^ID 1314^, (298)^ID 1315^,	-	-	(0.0028, IQY)^ID 1283, 1303^, (0.0027, IQY)^ID 1298, 1333, 1335^,	-	-	-
(388)^ID 1329^, (298)^ID 1330^, (388)^ID 1331^, (356)^ID 1332^, (376)^ID 1333^, (384)^ID 1334^, (376)^ID 1335^, (304)^ID 1336^, (343)^ID 1337^, (392)^ID 1338^, (234)^ID 1339^, (301,pr)^ID 1349^, (346,pr)^ID 1350^, (350)^ID 1351^, (275)^ID 1352^, (281,f)^ID 1354^, (304,f)^ID 1355^	-	-	(0.0026, IQY)^ID 1329, 1331, 1334, 1372, 1373^, (0.0028, IQY)^ID 1332^,	-	-	-
(389)^ID 1372^, (391)^ID 1373^, (212)^ID 1374^, (304)^ID 1375^, (238)^ID 1376^, (298)^ID 1377^, (303)^ID 1378^, (279)^ID 1379^, (290)^ID 1380^, (302)^ID 1381^, (321)^ID 1382^, (348)^ID 1383^, (297)^ID 1384^, (340)^ID 1385^, (261,f)^ID 1386^, (238,f)^ID 1387^, (299,f)^ID 1388^, (313,f)^ID 1389^, (329,f)^ID 1390^, (343,f)^ID 1391^, (371,f)^ID 1392^, (388,f)^ID 1393^, (343)^ID 1394^, (365)^ID 1395^, (381,pr,f)^ID 1433^ Protein ***** (30,f)^ID 1770^	(0.0029, YG)^ID 1394^, (0.0027, YG)^ID 1395^	-	(0.0047, IQY)^ID 1374^	-	-	-
^I^ Enzyme inhibitors (10^T^) (145,pr)^ID 1286, 1287^, (168,pr)^ID 1288^, (143,pr)^ID 1289^, (124)^ID 1290, 1293^, (123)^ID 1291^, (121,pr)^ID 1294, 1295^ Protein ***** (44,f)^ID 1292^	(0.0081, YG)^ID 1291^	-	(0.0060, YVL)^ID 1288^	-	-	-
^I^ Thaumatin-like proteins (3^T^) (173,pr)^ID 1296^, (154)^ID 1370^, (173)^ID 1371^	-	-	-	-	-	-
^I,VI^ Other (13^T^) (126,pr,f)^ID 1171^, (136,pr)^ID 1173, 1174^, isomerase (253)^ID 1766^, agglutinin isolectin (186,pr,f)^ID 1767^, (213,pr)^ID 1768^, (212,pr)^ID 1769^, (118)^ID 1771^ Proteins ***** (47)^ID 1132, 1172^, (18,f)^ID 1341^, (90,f)^ID 1432^, (27,f)^ID 1765^	(0.0079, YG, YGG)^ID 1766^, (0.0108, 2xYG)^ID 1767^, (0.0094, 2xYG)^ID 1768, 1769^, (0.0085, YG)^ID 1771^	(0.0074, GVM)^ID 1173, 1174^, (0.0040, GFL)^ID 1766^	-	-	-	(0.0079, 2xVVV)^ID 1766^

ImmD—immunomodulatory, ImmS—immunostymulatory, AntiB—antibacterial, AntiF—antifungal, AntiV—antiviral, AntiC—anticarcinogenic activities, ^T^—the number of total proteins including all protein fragments available from the BIOPEP-UWM database (accessed on August/September 2022 and August/September 2023. The data were updated as of 1 October 2023), pr—precursor, f—fragment of protein, * NAAR—the number of amino acid residues found in the analyzed protein or protein fragment, ** ID—identification number in the BIOPEP-UWM database, *** A—the A parameter (the frequency of bioactive fragment occurrence in a protein sequence, defined in the “Materials and Methods” section), **** seq—sequence of biopeptides, amino acid sequences presented in the form of a single-letter amino acid code: R—arginine, Q—glutamine, E—glutamic acid, G—glycine, I—isoleucine, L—leucine, M—methionine, F—phenylalanine, Y—tyrosine, V—valine, ***** Protein(s)—a protein or a protein fragment not meeting the molecular criterion, i.e., possessing at least 100 amino acid residues. HMW—high molecular weight, LMW—low molecular weight.

**Table A2:** Characteristics of the selected activities of rice (^I^ *Oryza sativa*, ^II^ *Oryza sativa [japonica cultivar-group]*, ^III^ *Oryza sativa* subsp. *japonica,* ^IV^ *Oryza sativa (indica cultivar-group)*) proteins.

Protein Groups (NAAR *)^ID^ **	ImmD (A ***, seq ****)^ID^ **	ImmS (A ***, seq ****)^ID^ **	AntiB (A ***, seq ****)^ID^ **	AntiF (A ***, seq ****)^ID^ **	AntiV (A ***, seq ****)^ID^ **	AntiC (A ***, seq ****)^ID^ **
^I^ Prolamins + globulins (11^T^) (149,pr)^ID 1152^, (149,pr)^ID 1153^, (148,pr)^ID 1154^, (150,pr)^ID 1155^, (134,pr)^ID 1168^, (156,pr)^ID 1550^, (134,pr)^ID 1580^, (101)^ID 1581^, (156)^ID 1592^, globulin-1 (174,f)^ID 1571^, globulin-2 (136,f)^ID 1572^	(0.0336, 3xYG, GIAASPFLQSAAFQLR, YGIYPR)^ID 1152^, (0.0338, 3xYG, GIAASPFLQSAAFQLR, YGIYPR)^ID 1154^, (0.0333, 3xYG, GIAASPFLQSAAFQLR, YGIYPR)^ID 1155^, (0.0099, YG)^ID 1581^	(0.0057, GVM)^ID 1571^	(0.0067, EIPT)^ID 1153^	-	-	-
^I^ Glutelins (16^T^) (499,pr)^ID 1535, 1537, 1539^, (498,pr)^ID 1536^, (500,pr)^ID 1538^, (496,pr)^ID 1540^, (510)^ID 1593^ Proteins ***** (24,pr,f)^ID 1582^, (60,f)^ID 1583, 1588^ (82,f)^ID 1584^, (72,f)^ID 1585^, (79,f)^ID 1586^, (83,f)^ID 1587^, (65,f)^ID 1589^, (46,f)^ID 1590^	(0.0040, YG, IAFKTNPNSMVSHIAGK)^ID 1535^, (0.0020, YG)^ID 1536, 1538^, (0.0040, YG, NSVFRALPVDVVANAYR)^ID 1537, 1539, 1540^	-	-	-	-	(0.0020, VVV)^ID 1535, 1593^
^I^ Oryzains + ^III^ expansin-B1 (4^T^) (471,pr)^ID 1547^, (458,pr)^ID 1548^, (362,pr)^ID 1549^, (267,pr)^ID 1772^	(0.0021, YG)^ID 1547^, (0.0066, 3xYG)^ID 1548^, (0.0055, 2xYG)^ID 1549^, (0.0075, 2xYG)^ID 1772^	(0.0021, EAE)^ID 1547^	-	-	-	(0.0037, VVV)^ID 1772^
^I^ Lectin + oleosins (3^T^) (227,pr)^ID 1533^, (148)^ID 1545^, (172)^ID 1546^	(0.0044, YG)^ID 1533^, (0.0068, YG)^ID 1545^	(0.0058, GFL)^ID 1546^	-	-	-	-
^I^ Glycine-rich proteins + other1 (9^T^) (165,pr)^ID 1541^, (183,pr)^ID 1542^, (162)^ID 1562^, (161)^ID 1563^, (135)^ID 1543^, (176)^ID 1544^, (177,pr)^ID 1560^, (139)^ID 1561^, (154)^ID 1564^	(0.0545, 9xYG)^ID 1541^, (0.0929, 15xYG, 2xYGG)^ID 1542^, (0.0988, 9xYG, 7xYGG)^ID 1562^, (0.0870, 9xYG, 5xYGG)^ID 1563^, (0.0074, GLF)^ID 1543^, (0.0114, YG, YGG)^ID 1544^, (0.0065, YG)^ID 1564^	(0.0055, LGY)^ID 1542^	(0.0057, YVL)^ID 1544^	-	-	(0.0057, VVV)^ID 1544^, (0.0056, VVV)^ID 1560^
^I,II^Allergenic proteins + ^I^ homolog protein + ^III^ profilin (13^T^) (166)^ID 1573^, (160,f)^ID 1574^, (157)^ID 1575^, (160)^ID 1576^, (111,f)^ID 1577^, (109,f)^ID 1578^, (113,f)^ID 1579^, (165,pr)^ID 1551^, (162,pr)^ID 1552^, (166,pr)^ID 1554^, (143)^ID 1534^, profilin A(131)^ID 1773^ Protein ***** (95,pr,f)^ID 1553^	(0.0063, GYPMYPLPR)^ID 1576^, (0.0076, GLF)^ID 1773^	(0.0060, LGY)^ID 1573, 1554^, (0.0063, LGY)^ID 1574^, (0.0090, LGY)^ID 1577^, (0.0092, LGY)^ID 1578^, (0.0088, LGY)^ID 1579^, (0.0061, LGY)^ID 1551^, (0.0062, LGY)^ID 1552^, (0.0070, EAE)^ID 1534^, (0.0076, GFL)^ID 1773^	(0.0070, IAK)^ID 1534^	-	-	(0.0127, 2xVVV)^ID 1575^
^I^ Tubulin chains (5^T^) (450)^ID 1555^, (444)^ID 1556^, (447)^ID 1557, 1558^, (469)^ID 1559^	(0.0022, YG)^ID 1555, 1557, 1558^	(0.0022, GFL)^ID 1555^, (0.0090, 4xEAE)^ID 1556^, (0.0045, 2xEAE)^ID 1557, 1558^	-	-	-	(0.0043, 2xVVV)^ID 1559^
^I^ Germin-like proteins (6^T^) (224)^ID 1565^, (225)^ID 1566^, (181,f)^ID 1567^, (216)^ID 1568^, (213)^ID 1569^, (225)^ID 1570^	(0.0044, TPRK)^ID 1570^	-	-	-	-	-
^I,III^ Enzymes + other2 (18^T^) (571)^ID 1699^, (958)^ID 1703^, (825,pr)^ID 1704^, (841)^ID 1705^, (820)^ID 1706^, (990)^ID 1713^, (986,pr)^ID 1714^, (941)^ID 1716^, (718)^ID 1717^, (736)^ID 1718^, (955)^ID 1719^, (948)^ID 1720^, (954)^ID 1721^, (942)^ID 1722^, (865)^ID 1723^, (943)^ID 1724^ Proteins ***** (74)^ID 1707, 1710^	(0.0021, 2xYG)^ID 1703^, (0.0061, 3xYG, KRP, GLF)^ID 1704^, (0.0071, 4xYG, KRP, GLF)^ID 1705^, (0.0040, 2xYG, IKRP, GLF)^ID 1713^, (0.0041, 2xYG, IKRP, GLF)^ID 1714^, (0.0027, GRKP, RKP)^ID 1718^, (0.0021, 2xYG)^ID 1719, 1722^_,_ (0.0011, YG)^ID 1720^, (0.0010, YG)^ID 1721^, (0.0012, YG)^ID 1723^, (0.0021, YG, YGG)^ID 1724^	(0.0010, LGY)^ID 1703^, (0.0012, LGY)^ID 1704, 1705^, (0.0011, GFL)^ID 1720^, (0.0010, GFL)^ID 1721^, (0.0011, LLY)^ID 1722^, (0.0035, 2xGFL, LLY)^ID 1723^	(0.0010, YVL)^ID 1713, 1714^, (0.0011, IAK)^ID 1716, 1724^, (0.0014, IQY)^ID 1717, 1718^, (0.0010, IAK)^ID 1719^, (0.0012, IAK)^ID 1723^	-	-	-
^I^ Actins + other3 (23^T^) (377)^ID 1529^, (379)^ID 1530^, (376)^ID 1531^, (376)^ID 1532^, (289)^ID 1685^, (464)^ID 1686^, (385,f)^ID 1687^, (345)^ID 1688^, (232)^ID 1689^, (286)^ID 1690^, (291)^ID 1691^, (331)^ID 1692^, (388)^ID 1693^, (356,f)^ID 1694^, (230)^ID 1695, 1696^, (353)^ID 1697^, (353)^ID 1698^, (477)^ID 1708^, (324)^ID 1709^, (514)^ID 1711^, (202)^ID 1712^, (486)^ID 1715^	(0.0027, YG)^ID 1529, 1531, 1532^, (0.0026, YG)^ID 1530, 1693^, (0.0069, 2xGLF)^ID 1685^, (0.0065, 3xYG)^ID 1686^, (0.0052, YG, GLF)^ID 1687^, (0.0058, YG, YGG)^ID 1688^, (0.0043, YG)^ID 1689^, (0.0035, GLF)^ID 1690^, (0.0034, GLF)^ID 1691^, (0.0030, YG)^ID 1692^, (0.0028, YG)^ID 1697, 1698^, (0.0042, 2xYG)^ID 1708^, (0.0031, YG)^ID 1709^, (0.0039, YG, RKP)^ID 1711^, (0.0021, YG)^ID 1715^	(0.0027, GVM)^ID 1529, 1531, 1532^, (0.0026, GVM)^ID 1530^, (0.0043, 2xGFL)^ID 1686^, (0.0078, LGY, 2xGFL)^ID 1687^, (0.0029, GFL)^ID 1688^, (0.0070, EAE, GVM)^ID 1690^, (0.0030, GFL)^ID 1692^, 0.0028, LLY)^ID 1697, 1698^, (0.0021, EAE)^ID 1708^, (0.0021, LLY)^ID 1715^	(0.0027, IAK)^ID 1529^, (0.0035, YVL)^ID 1690^, (0.0030, IAK)^ID 1692^	-	(0.0027, ERF)^ID 1529, 1532^, (0.0026, ERF)^ID 1530^, (0.0043, ERF)^ID 1695, 1696^	(0.0026, VVV)^ID 1687^, (0.0043, VVV)^ID 1689^, (0.0035, VVV)^ID 1690^
^I,IV^ Other4 (4^T^) (342)^ID 1591^, (318,pr)^ID 1700^, Proteins ***** (93)^ID 1701^, (86)^ID 1702^	(0.0058, 2xYG)^ID 1591^	-	-	-	-	-

ImmD—immunomodulatory, ImmS—immunostymulatory, AntiB—antibacterial, AntiF—antifungal, AntiV—antiviral, AntiC—anticarcinogenic activities, ^T^—the number of total proteins including all protein fragments available from the BIOPEP-UWM database (accessed on August/September 2022 and August/September 2023. The data were updated as of 1 October 2023), pr—precursor, f—fragment of protein, * NAAR—the number of amino acid residues found in the analyzed protein or protein fragment, ** ID—identification number in the BIOPEP-UWM database, *** A—the A parameter (the frequency of bioactive fragment occurrence in a protein sequence, defined in the “Materials and Methods” section), **** seq—sequence of biopeptides, amino acid sequences presented in the form of a single-letter amino acid code: A—alanine, R—arginine, N—asparagine, D—aspartic acid, Q—glutamine, E—glutamic acid, G—glycine, H—histidine, I—isoleucine, L—leucine, K—lysine, M—methionine, F—phenylalanine, P—proline, S—serine, T—threonine, W—tryptophan, Y—tyrosine, V—valine, ***** Protein(s)—a protein or a protein fragment not meeting the molecular criterion, i.e., possessing at least 100 amino acid residues.

**Table A3:** Characteristics of the selected activities of barley (^I^ *Hordeum vulgare*) proteins.

Protein Groups (NAAR *)^ID^ **	ImmD (A ***, seq ****)^ID^	ImmS (A ***, seq ****)^ID^	AntiB (A ***, seq ****)^ID^	AntiF (A ***, seq ****)^ID^	AntiV (A ***, seq ****)^ID^	AntiC (A ***, seq ****)^ID^
^I^ Hordeins (16^T^) (290,pr)^ID 1623^, (293,pr)^ID 1603^, (264,f)^ID 1604^, (105,f)^ID 1605^, (310)^ID 1627^, (475,f)^ID 1625, 1626^, (757)^ID 1632^, (305,pr)^ID 1150^, (289)^ID 1602^, (255,f)^ID 1628^ Proteins ***** (72,f)^ID 1606^, (68,f)^ID 1607^, (50,f)^ID 1624^, (40)^ID 1629^, (35)^ID 1630^	(0.0013, YG)^ID 1632^	-	(0.0034, IQY)^ID 1603^	-	-	-
^I^ Globulin + hordothionins + other1 (10^T^) (224)^ID 1634^, (127,pr)^ID 1176^, (136,pr)^ID 1621^, (179,f)^ID 1622^, (499)^ID 1633^, (146)^ID 1645^, (137,f)^ID 1646^, profilin-1 (131)^ID 1764^ Proteins ***** (31,f)^ID 1656^, (47,f)^ID 1175^	(0.0045, YG)^ID 1634^, (0.0056, RKP)^ID 1622^, (0.0020, YG)^ID 1633^, (0.0068, YG)^ID 1645^, (0.0076, GLF)^ID 1764^	(0.0074, GVM)^ID 1621^, (0.0060, 2xEAE, LGY)^ID 1633^	-	-	-	(0.0076, VWV)^ID 1764^
^I^ Tubulin chains (4^T^) (450)^ID 1616^, (451)^ID 1617, 1618^, (447)^ID 1619^	(0.0022, YG)^ID 1616–1619^	(0.0022, GFL)^ID 1616–1618^, (0.0067, 3xEAE)^ID 1619^	-	-	-	-
^I^ Thaumatin-like proteins (4^T^) (233)^ID 1647^, (227)^ID 1648^, (226)^ID 1649^, (171)^ID 1650^	(0.0043, YG)^ID 1647^	-	-	-	-	-
^I^ Hordoindolines (14^T^) (142,f)^ID 1635, 1636, 1639–1644, 1653–1655^, (103,f)^ID 1637, 1638^, (141,f)^ID 1652^	(0.0070, YG)^ID 1635, 1636, 1639–1644^	-	-	-	-	-
^I^ Enzyme inhibitors + other2 (11^T^) (146,pr,f)^ID 1608^, (152,pr)^ID 1609^, (145,pr)^ID 1610^, (149,pr)^ID 1611^, (171,pr)^ID 1613^, (203,pr)^ID 1614^, (147,pr)^ID 1615^, (103,f)^ID 1631^, (245)^ID 1651^ Proteins ***** (35,f)^ID 1612^, (44,f)^ID 1620^	(0.0049, YG)^ID 1614^, (0.0097, YG)^ID 1631^	(0.0041, LLY)^ID 1651^	(0.0058, YVL)^ID 1613^, (0.0049, YVL)^ID 1614^, (0.0097, YVL)^ID 1631^	-	-	(0.0049, VVV)^ID 1614^

ImmD—immunomodulatory, ImmS—immunostymulatory, AntiB—antibacterial, AntiF—antifungal, AntiV—antiviral, AntiC—anticarcinogenic activities, ^T^—the number of total proteins including all protein fragments available from the BIOPEP-UWM database (accessed on August/September 2022 and August/September 2023. The data were updated as of 1 October 2023), pr—precursor, f—fragment of protein, * NAAR—the number of amino acid residues found in the analyzed protein or protein fragment, ** ID—identification number in the BIOPEP-UWM database, *** A—the A parameter (the frequency of bioactive fragment occurrence in a protein sequence, defined in the “Materials and Methods” section), **** seq—sequence of biopeptides, amino acid sequences presented in the form of a single-letter amino acid code: A—alanine, R—arginine, Q—glutamine, E—glutamic acid, G—glycine, I—isoleucine, L—leucine, K—lysine, M—methionine, F—phenylalanine, P—proline, W—tryptophan, Y—tyrosine, V—valine, ***** Protein(s)—a protein or a protein fragment not meeting the molecular criterion, i.e., possessing at least 100 amino acid residues.

**Table A4:** Characteristics of the selected activities of oat (^I^ *Avena sativa*) proteins.

Protein Groups (NAAR *)^ID^ **	ImmD (A ***, seq ****)^ID^ **	ImmS (A ***, seq ****)^ID^ **	AntiB (A ***, seq ****)^ID^ **	AntiF (A ***, seq ****)^ID^ **	AntiV (A ***, seq ****)^ID^ **	AntiC (A ***, seq ****)^ID^ **
^I^ 12S + 11S globulins (5^T^) (518,pr)^ID 1165^, (494)^ID 1167^, (313,f)^ID 1465^, (551,pr)^ID 1463^, (527)^ID 1464^	-	-	-	-	-	-
^I^ Avenins (9^T^) (220,pr)^ID 1451^, (214,pr)^ID 1452^, (181,pr)^ID 1457^, (222,pr)^ID 1458^, (182)^ID 1462^ Proteins ***** (29,f)^ID 1450^, (36,f)^ID 1459^, (52,f)^ID 1460^, (43,f)^ID 1461^	-	-	(0.0047, IAK)^ID 1452^	-	-	-
^I^ Thaumatin-like pathogenesis-related proteins (4^T^) (169,pr)^ID 1453–1456^	-	-	-	-	-	-
^I^ Avenoindolines + other (5^T^) (148,pr)^ID 1466, 1467^ Proteins ***** (27,f)^ID 1447^, (19,f)^ID 1448^, (23,f)^ID 1449^	-	-	-	-	-	-

ImmD—immunomodulatory, ImmS—immunostymulatory, AntiB—antibacterial, AntiF—antifungal, AntiV—antiviral, AntiC—anticarcinogenic activities, ^T^—the number of total proteins including all protein fragments available from the BIOPEP-UWM database (accessed on August/September 2022 and August/September 2023. The data were updated as of 1 October 2023), pr—precursor, f—fragment of protein, * NAAR—the number of amino acid residues found in the analyzed protein or protein fragment, ** ID—identification number in the BIOPEP-UWM database, *** A—the A parameter (the frequency of bioactive fragment occurrence in a protein sequence, defined in the “Materials and Methods” section), **** seq—sequence of biopeptides, amino acid sequences presented in the form of a single-letter amino acid code: A—alanine, I—isoleucine, K—lysine, ***** Proteins—a protein or a protein fragment not meeting the molecular criterion, i.e., possessing at least 100 amino acid residues.

**Table A5:** Characteristics of the selected activities of buckwheat (^I^ *Fagopyrum esculentum*, ^II ^*Fagopyrum gracilipes*, ^III ^*Fagopyrum tataricum*), rye (^IV^ *Secale cereale*), sorghum (^V^ *Sorghum vulgare*), and maize (^VI^ *Zea mays*) proteins.

Protein Groups (NAAR *)^ID^ **	ImmD (A ***, seq ****)^ID^ **	ImmS (A ***, seq ****)^ID^ **	AntiB (A ***, seq ****)^ID^ **	AntiF (A ***, seq ****)^ID^ **	AntiV (A ***, seq ****)^ID^ **	AntiC (A ***, seq ****)^ID^ **
^I^ 13S globulins+ ^II^ seed storage protein (6 ^I^) (565,pr)^ID 1468^, (504,pr)^ID 1469^, (538,pr)^ID 1470^, (194)^ID 1471^, (453,f)^ID 1475^, (191)^ID 1480^	(0.0052, YG)^ID 1471^, (0.0022, GLF)^ID 1475^	(0.0103, GFL, EAE)^ID 1471^	(0.0022, YVL)^ID 1475^	-	(0.0018, ERF)^ID 1468^, (0.0020, ERF)^ID 1469^, (0.0019, ERF)^ID 1470^, (0.0052, ERF)^ID 1471^_,_ ^1480^	(0.0018, VVV)^ID 1468,^ (0.0019, VVV)^ID 1470^, (0.0022, VVV)^ID 1475^
^I^ Legumin-like 13S+ legumin-type protein (2^T^) (160,f)^ID 1474^, (303,f)^ID 1476^	-	(0.0033, GVM)^ID 1476^	-	-	-	(0.0033, VVV)^ID 1476^
^I^ Vicilin-like protein (1^T^) (140)^ID 1473^	-	-	-	-	-	-
^I,III^ Allergenic protein+ other1 (4^T^) (195,f)^ID 1477^, (133)^ID 1478^, (173)^ID 1479^, (252)^ID 1472^	(0.0079, 2xYG)^ID 1472^	(0.0040, GVM)^ID 1472^	-	-	(0.0051, ERF)^ID 1477^	(0.0051, VVV)^ID 1477^
^IV ^Omega-secalin+ other2 (14^T^) (357)^ID 1595^ Proteins ***** (23,f)^ID 1439, 1440, 1446, 1597, 1601^, (16,f)^ID 1441^, (34,f)^ID 1442^, (24,f)^ID 1443^, (29,f)^ID 1444^, (20,f)^ID 1445^, (25,f)^ID 1594^, (32,f)^ID 1596^, (16,f)^ID 1598^	-	-	-	-	-	(0.0056, 2xVVV)^ID 1595^
^IV ^Glutenin, HMW subunits (2^T^) (754,pr)^ID 1599^, (713,pr)^ID 1600^	-	-	-	-	-	(0.0013, VVV)^ID 1599^
^V ^Kafirins (3^T^) (269,pr)^ID 1149^, (267,pr)^ID 1196, 1197^	(0.0037, PFNQL)^ID 1196^	-	-	-	-	-
^VI ^Legumin 1 + non-specific lipid transfer protein (2^T^) (483)^ID 1156^, (120,pr)^ID 1774^	(0.0104, 5xYG)^ID 1156^	-	-	-	-	(0.0021, VVV)^ID 1156^

ImmD—immunomodulatory, ImmS—immunostymulatory, AntiB—antibacterial, AntiF—antifungal, AntiV—antiviral, AntiC—anticarcinogenic activities, ^T^—the number of total proteins including all protein fragments available from the BIOPEP-UWM database (accessed on August/September 2022 and August/September 2023. The data were updated as of 1 October 2023), pr—precursor, f—fragment of protein, * NAAR—the number of amino acid residues found in the analyzed protein or protein fragment, ** ID—identification number in the BIOPEP-UWM database, *** A—the A parameter (the frequency of bioactive fragment occurrence in a protein sequence, defined in the “Materials and Methods” section), **** seq—sequence of biopeptides, amino acid sequences presented in the form of a single-letter amino acid code: A—alanine, R—arginine, N—asparagine, Q—glutamine, E—glutamic acid, G—glycine, L—leucine, M—methionine, F—phenylalanine, P—proline, Y—tyrosine, V—valine, ***** Proteins—a protein or a protein fragment not meeting the molecular criterion, i.e., possessing at least 100 amino acid residues.

**Table A6:** Characteristics of the selected activities of garden pea (^I^ *Pisum sativum*) proteins.

Protein Groups (NAAR *)^ID^ **	ImmD (A***, seq****)^ID^**	ImmS (A***, seq****)^ID^**	AntiB (A***, seq****)^ID^**	AntiF (A***, seq****)^ID^**	AntiV (A***, seq****)^ID^**	AntiC (A***, seq****)^ID^**
^I^ Albumins (7^T^) (130,pr)^ID 1484–1489^, (231)^ID 1490^	(0.0077, YG)^ID 1484, 1485, 1489^, (0.0154, 2xYG)^ID 1486^, (0.0154, YG, GLF)^ID 1487, 1488^, (0.0087, 2xYG)^ID 1490^	(0.0077, EAE)^ID 1484–1489^	(0.0043, YVL)^ID 1490^	-	-	-
^I^ Legumins (7^T^) (498)^ID 1139^, (481)^ID 1144^, (517,pr)^ID 1502^, (338,f)^ID 1503^, (350,f)^ID 1157^, (566,pr)^ID 1512^ Protein ***** (70,f)^ID 1521^	-	(0.0021, LLY)^ID 1144^, (0.0059, 2xLLY)^ID 1503^, (0.0029, LLY)^ID 1157^, (0.0035, 2xLLY)^ID 1512^	(0.0030, YVL)^ID 1503^, (0.0018, YVL)^ID 1512^	-	-	(0.0021, VVV)^ID 1144^, (0.0030, VVV)^ID 1503^, (0.0029, VVV)^ID 1157^, (0.0018, VVV)^ID 1512^
^I^ Convicilins (2^T^) (613,pr)^ID 1524^, (571,pr)^ID 1491^ /Provicilins (2^T^) (277,f)^ID 1510^, (410,f)^ID 1511^	(0.0036, YG)^ID 1510^	-	-	-	(0.0024, LLEY)^ID 1511^	-
^I^ Vicilins (5^T^) (124)^ID 1237^, (432)^ID 1238^, (415,f)^ID 1518, 1519^, (156,f)^ID 1523^	-	-	-	-	(0.0023, LLEY)^ID 1238^, (0.0024, LLEY)^ID 1518, 1519^, (0.0064, LLEY)^ID 1523^	-
^I^ Tubulins (4^T^) (452)^ID 1506^, (450)^ID 1507^, (440,f)^ID 1508^, (447,f)^ID 1509^	(0.0022, YG)^ID 1506^, (0.0067, 3xYG)^ID 1507^, (0.0023, YG)^ID 1508^, (0.0067, 2xYG, YGG)^ID 1509^	(0.0022, GFL)^ID 1506^, (0.0044, 2xEAE)^ID 1507^, (0.0045, 2xEAE)^ID 1508, 1509^	-	-	-	-
^I^ Actins (3^T^) (376)^ID 1481, 1482^, (377)^ID 1483^	(0.0027, YG)^ID 1481–1483^	(0.0027, GVM)^ID 1481, 1482^, (0.0053, EAE, GVM)^ID 1483^	(0.0027, IAK)^ID 1483^	-	(0.0027, ERF)^ID 1481–1483^	-
^I^ Histones (5^T^) (150)^ID 1497^, (149)^ID 1498^, (135)^ID 1499^, (102)^ID 1500^, (251)^ID 1517^ Lectin (1^T^) (275,pr)^ID 1501^	(0.0133, YG, YGG)^ID 1497^, (0.0201, YG, YGG, KTPK)^ID 1498^, (0.0074, GLF)^ID 1499^, (0.0196, YG, DNIQGITKPAIR) ^ID 1500^, (0.0040, TPRK)^ID 1517^	-	(0.0040, IAK)^ID 1517^	-	-	-
^I^ Disease resistance response proteins (5^T^) (159)^ID 1494^, (158)^ID 1495, 1496^ Proteins ***** (72,pr)^ID 1492^, (74,pr)^ID 1493^	(0.0063, GLF)^ID 1494, 1495^	-	(0.0063, IAK)^ID 1494, 1495^, (0.0127, YVL, IAK)^ID 1496^	-	-	-
^I^ Hypothetical proteins (2^T^) (562)^ID 1515, 1516^ Proteins ***** Nodulins (2^T^) (61,pr,f)^ID 1504^, (47,f)^ID 1520^	(0.0036, 2xYG)^ID 1515, 1516^	(0.0036, 2xEAE)^ID 1515, 1516^	-	-	-	-
^I^ Germin-like proteins (2^T^) (195,f)^ID 1526, 1527^	(0.0051, YG)^ID 1526, 1527^	-	-	-	-	-
^I^ Others (6^T^) (135)^ID 1505^, ubiquitin (457)^ID 1513^, ubiquitin (381)^ID 1525^, (150)^ID 1514^, (533)^ID 1522^, proline-rich protein (214)^ID 1528^	(0.0074, YG)^ID 1505^, (0.0067, GLF)^ID 1514^, (0.0094, 3xYG, YGG, GLF)^ID 1522^	(0.0019, EAE)^ID 1522^	-	-	(0.0074, ERF)^ID 1505^	-

ImmD—immunomodulatory, ImmS—immunostymulatory, AntiB—antibacterial, AntiF—antifungal, AntiV—antiviral, AntiC—anticarcinogenic activities, ^T^—the number of total proteins including all protein fragments available from the BIOPEP-UWM database (accessed on August/September 2022 and August/September 2023. The data were updated as of 1 October 2023), pr—precursor, f—fragment of protein, * NAAR—the number of amino acid residues found in the analyzed protein or protein fragment, ** ID—identification number in the BIOPEP-UWM database, *** A—the A parameter (the frequency of bioactive fragment occurrence in a protein sequence, defined in the “Materials and Methods” section), **** seq—sequence of biopeptides, amino acid sequences presented in the form of a single-letter amino acid code: A—alanine, R—arginine, N—asparagine, D—aspartic acid, Q—glutamine, E—glutamic acid, G—glycine, I—isoleucine, L—leucine, K—lysine, M—methionine, F—phenylalanine, P—proline, T—threonine, W—tryptophan, Y—tyrosine, V—valine, ***** Protein(s)—a protein or a protein fragment not meeting the molecular criterion, i.e., possessing at least 100 amino acid residues.

**Table A7:** Characteristics of the selected activities of kidney/French bean (^I^ *Phaseolus vulgaris*) and runner bean (^II^ *Phaseolus coccineus*) proteins.

Protein Groups (NAAR *)^ID^ **	ImmD (A ***, seq ****)^ID^ **	ImmS (A ***, seq ****)^ID^**	AntiB (A ***, seq ****)^ID^ **	AntiF (A ***, seq ****)^ID^ **	AntiV (A ***, seq ****)^ID^ **	AntiC (A ***, seq ****)^ID^ **
^I^ Phaseolins (3^T^) (397)^ID 1151^, (436,pr)^ID 1241^, (421,pr)^ID 1242^	(0.0025, YG)^ID 1151^, (0.0023, YG)^ID 1241^, (0.0024, YG)^ID 1242^	-	-	-	-	-
^I^ Lectin + ^I,II^ phytohemagglutinins (4^T^) (275,pr)^ID 1243^, (273,pr)^ID 1244^, (275,pr)^ID 1245^, (273,pr)^ID 1247^	(0.0036, YG)^ID 1243^, (0.0037, GLF)^ID 1244^, (0.0036, GLF)^ID 1245^, (0.0037, GLF)^ID 1247^	(0.0037, GFL)^ID 1244, 1247^	-	-	-	-
^I^ Arcelin + ^II^ Kunitz trypsin inhibitor (2^T^) (266,pr)^ID 1246^ Protein ***** (73)^ID 1248^	(0.0038, GLF)^ID 1246^	-	-	-	-	-

ImmD—immunomodulatory, ImmS—immunostymulatory, AntiB—antibacterial, AntiF—antifungal, AntiV—antiviral, AntiC—anticarcinogenic activities, ^T^—the number of total proteins including all protein fragments available from the BIOPEP-UWM database (accessed on August/September 2022 and August/September 2023. The data were updated as of 1 October 2023), pr—precursor, f—fragment of protein, * NAAR—the number of amino acid residues found in the analyzed protein or protein fragment, ** ID—identification number in the BIOPEP-UWM database, *** A—the A parameter (the frequency of bioactive fragment occurrence in a protein sequence, defined in the “Materials and Methods” section), **** seq—sequence of biopeptides, amino acid sequences presented in the form of a single-letter amino acid code: G—glycine, L—leucine, F—phenylalanine, Y—tyrosine, ***** Protein(s)—a protein or a protein fragment not meeting the molecular criterion, i.e., possessing at least 100 amino acid residues.

**Table A8:** Characteristics of selected activities of soybean (^I^ *Glycine max*) proteins.

Protein Groups (NAAR *)^ID^ **	ImmD (A ***, seq ****)^ID^ **	ImmS (A ***, seq ****)^ID^ **	AntiB (A ***, seq ****)^ID^ **	AntiF (A ***, seq ****)^ID^ **	AntiV (A ***, seq ****)^ID^ **	AntiC (A ***, seq ****)^ID^ **
^I^ Albumin 1 (1^T^) (156,pr)^ID 1160^	-	-	-	-	-	-
^I^ (Basic) 7S globulin (1^T^) (427,pr)^ID 1162^	(0.0023, TKPI)^ID 1162^	(0.0023, GVM)^ID 1162^	-	-	-	-
^I^ Beta-conglycinins (alpha chain) (2^T^)/ (639,pr)^ID 1239^, (605,pr)^ID 1775^ ^I^ G4 glycinin (1^T^) (562,pr)^ID 1240^	(0.0016, YG)^ID 1239^, (0.0017, YG)^ID 1775^	-	-	-	-	(0.0017, VVV)^ID 1775^
^I^ P34 probable thiol protease (1^T^) (379,pr)^ID 1776^	(0.0053, 2xYG)^ID 1776^	(0.0026, GFL)^ID 1776^	(0.0026, IAK)^ID 1776^	-	-	-
^I^ Profilin-1 (1^T^) (131)^ID 1777^ ^I^ Stress-induced protein SAM22 (1^T^) (158)^ID 1778^	-	(0.0063, LGY)^ID 1778^	-	-	-	-

ImmD—immunomodulatory, ImmS—immunostymulatory, AntiB—antibacterial, AntiF—antifungal, AntiV—antiviral, AntiC—anticarcinogenic activities, ^T^—the number of total proteins including all protein fragments available from the BIOPEP-UWM database (accessed on August/September 2022 and August/September 2023. The data were updated as of 1 October 2023), pr—precursor, f—fragment of protein, * NAAR—the number of amino acid residues found in the analyzed protein or protein fragment, ** ID—identification number in the BIOPEP-UWM database, *** A—the A parameter (the frequency of bioactive fragment occurrence in a protein sequence, defined in the “Materials and Methods” section), **** seq—sequence of biopeptides, amino acid sequences presented in the form of a single-letter amino acid code: A—alanine, G—glycine, I—isoleucine, L—leucine, K—lysine, M—methionine, F—phenylalanine, P—proline, T—threonine, Y—tyrosine, V—valine.

**Table A9:** Characteristics of selected activities of broad bean (^I^ *Vicia faba*), (^II^ *Vicia narbonensis*, ^III^ *Vicia pannonica*), lentil (^IV^ *Lens culinaris* ssp*. orientalis*), and white lupine (^V^ *Lupinus albus*) proteins.

Protein Groups (NAAR *)^ID^ **	ImmD (A ***, seq ****)^ID^ **	ImmS (A ***, seq ****)^ID^ **	AntiB (A ***, seq ****)^ID^ **	AntiF (A ***, seq ****)^ID^ **	AntiV (A ***, seq ****)^ID^ **	AntiC (A ***, seq ****)^ID^ **
^I^ Legumin (chain B) (1^T^) (329,f)^ID 1142^/ ^I^ Legumins, HMW (2^T^) (136,f)^ID 1158^, (547)^ID 1159^	(0.0074, YG)^ID 1158^, (0.0018, YG)^ID 1159^	(0.0030, LLY)^ID 1142^, (0.0074, EAE)^ID 1158^, (0.0055, 2xLLY, EAE)^ID 1159^	(0.0018, YVL)^ID 1159^	-	-	(0.0030, VVV)^ID 1142^, (0.0018, VVV)^ID 1159^
^II,III^ Narbonins (2S proteins, seed storage proteins) (2^T^) (290)^ID 1190^, (291)^ID 1191^	-	-	-	-	-	(0.0034, VWV)^ID 1190, 1191^
^IV ^Convicilin (1^T^) (162,f)^ID 1798^	-	-	-	-	-	-
^V ^PR-10 protein (1^T^) (158)^ID 1192^	-	(0.0063, LGY)^ID 1192^	(0.0063, YVL)^ID 1192^	-	-	-

ImmD—immunomodulatory, ImmS—immunostymulatory, AntiB—antibacterial, AntiF—antifungal, AntiV—antiviral, AntiC—anticarcinogenic activities, ^T^—the number of total proteins including all protein fragments available from the BIOPEP-UWM database (accessed on August/September 2022 and August/September 2023. The data were updated as of 1 October 2023), f—fragment of protein, * NAAR—the number of amino acid residues found in the analyzed protein or protein fragment, ** ID—identification number in the BIOPEP-UWM database, *** A—the A parameter (the frequency of bioactive fragment occurrence in a protein sequence, defined in the “Materials and Methods” section), **** seq—sequence of biopeptides, amino acid sequences presented in the form of a single-letter amino acid code: A—alanine, E—glutamic acid, G—glycine, L—leucine, W—tryptophan, Y—tyrosine, V—valine. HMW—high molecular weight.

**Table A10:** Characteristics of the selected activities of peanut (^I^ *Arachis hypogaea*) proteins.

Protein Groups (NAAR *)^ID^ **	ImmD (A ***, seq ****)^ID^ **	ImmS (A***, seq ****)^ID^ **	AntiB (A ***, seq ****)^ID^ **	AntiF (A ***, seq ****)^ID^ **	AntiV (A ***, seq ****)^ID^ **	AntiC (A ***, seq)^ID^ **
^I^ Glycinins (2^T^) (507,f)^ID 1781^, (530)^ID 1782^	(0.0039, 2xYG)^ID 1781^, (0.0038, 2xYG)^ID 1782^	-	-	-	-	-
^I^ Allergen Ara h 1 (clone P17) (1^T^) (614,pr)^ID 1779^ ^I^ Arachin 6 (1^T^) (529)^ID 1785^	(0.0016, YG)^ID 1779^, (0.0038, 2xYG)^ID 1785^	-	-	-	-	(0.0016, VVV)^ID 1779^
^I^ Conglutins (conglutin-7, conglutin) (2^T^) (172,pr)^ID 1780^, (145,pr)^ID 1784^	(0.0058, YG)^ID 1780^	-	-	-	-	-
^I^ Profilin (1^T^) (131)^ID 1783^	-	-	-	-	-	-

ImmD—immunomodulatory, ImmS—immunostymulatory, AntiB—antibacterial, AntiF—antifungal, AntiV—antiviral, AntiC—anticarcinogenic activities, ^T^—the number of total proteins including all protein fragments available from the BIOPEP-UWM database (accessed on August/September 2022 and August/September 2023. The data were updated as of 1 October 2023), pr—precursor, f—fragment of protein, * NAAR—the number of amino acid residues found in the analyzed protein or protein fragment, ** ID—identification number in the BIOPEP-UWM database, *** A—the A parameter (the frequency of bioactive fragment occurrence in a protein sequence, defined in the “Materials and Methods” section), **** seq—sequence of biopeptide, amino acid sequences presented in the form of a single-letter amino acid code: G—glycine, Y—tyrosine, V—valine.

**Table A11:** Characteristics of selected activities of oriental sesame (^I^ *Sesamum indicum*) proteins.

Protein Groups (NAAR *)^ID^ **	ImmD (A ***, seq ****)^ID^ **	ImmS (A ***, seq ****)^ID^ **	AntiB (A ***, seq ****)^ID^ **	AntiF (A ***, seq ****)^ID^ **	AntiV (A ***, seq ****)^ID^ **	AntiC (A ***, seq ****)^ID^ **
^I^ Oleosins (2^T^) (168)^ID 1788^, (145)^ID 1789^	(0.0069, YG)^ID 1789^	(0.0069, GFL)^ID 1789^	-	-	-	-
^I^ 2S seed storage protein 1 (1^T^) (148,pr)^ID 1786^	(0.0069, YG)^ID 1786^	-	-	-	-	-
^I^ 7S globulin (1^T^) (585)^ID 1787^	(0.0017, YG)^ID 1787^	(0.0017, EAE)^ID 1787^	-	-	-	-
^I^ 11S globulin seed storage protein 2 (1^T^) (459,pr)^ID 1790^	-	-	-	-	-	-

ImmD—immunomodulatory, ImmS—immunostymulatory, AntiB—antibacterial, AntiF—antifungal, AntiV—antiviral, AntiC—anticarcinogenic activities, ^T^—the number of total proteins including all protein fragments available from the BIOPEP-UWM database (accessed on August/September 2022 and August/September 2023. The data were updated as of 1 October 2023), pr—precursor, * NAAR—the number of amino acid residues found in the analyzed protein or protein fragment, ** ID—identification number in the BIOPEP-UWM database, *** A—the A parameter (the frequency of bioactive fragment occurrence in a protein sequence, defined in the “Materials and Methods” section), **** seq—sequence of biopeptides, amino acid sequences presented in the form of a single-letter amino acid code: A—alanine, E—glutamic acid, G—glycine, L—leucine, F—phenylalanine, Y—tyrosine.

**Table A12:** Characteristics of the selected activities of celery (^I^ *Apium graveolens*) proteins.

Protein Groups (NAAR *)^ID^ **	ImmD (A ***, seq ****)^ID^ **	ImmS (A ***, seq ****)^ID^ **	AntiB (A ***, seq ****)^ID^ **	AntiF (A ***, seq ****)^ID^ **	AntiV (A ***, seq ****)^ID^ **	AntiC (A ***, seq ****)^ID^ **
^I^ Chlorophyll a-b binding protein, chloroplast (1^T^) (264,pr)^ID 1757^	(0.0076, 2xYG)^ID 1757^	-	-	-	-	-
^I^ Major allergen(s) Api g 1, 2 (3^T^) (154)^ID 1791^, (159)^ID 1792^ Protein ***** (86,f)^ID 1755^	-	(0.0063, GFL)^ID 1792^	-	-	-	(0.0063, VVV)^ID 1792^
^I^ Profilin (1^T^) (134)^ID 1756^	-	-	-	-	-	-

ImmD—immunomodulatory, ImmS—immunostymulatory, AntiB—antibacterial, AntiF—antifungal, AntiV—antiviral, AntiC—anticarcinogenic activities, ^T^—the number of total proteins including all protein fragments available from the BIOPEP-UWM database (accessed on August/September 2022 and August/September 2023. The data were updated as of 1 October 2023), pr—precursor, f—fragment of protein, * NAAR—the number of amino acid residues found in the analyzed protein or protein fragment, ** ID—identification number in the BIOPEP-UWM database, *** A—the A parameter (the frequency of bioactive fragment occurrence in a protein sequence, defined in the “Materials and Methods” section), **** seq—sequence of biopeptides, amino acid sequences presented in the form of a single-letter amino acid code: G—glycine, L—leucine, F—phenylalanine, Y—tyrosine, V—valine, ***** Protein—a protein or a protein fragment not meeting the molecular criterion, i.e., possessing at least 100 amino acid residues.

**Table A13:** Characteristics of selected activities of mouse-ear cress (^I^ *Arabidopsis thaliana*), cocoa (^II^ *Theobroma cacao*), ginkgo (^III^ *Ginkgo biloba*), pumpkin (^IV^ *Cucurbita species*), common sunflower (^V^ *Helianthus annuus*), upland cotton (^VI^ *Gossypium hirsutum*), rape (^VII^ *Brassica napus*), and serendipity berry (^VIII^ *Dioscoreophyllum cumminsii*) proteins.

Protein Groups (NAAR *)^ID^ **	ImmD (A ***, seq ****)^ID^ **	ImmS (A ***, seq ****)^ID^ **	AntiB (A ***, seq ****)^ID^ **	AntiF (A ***, seq ****)^ID^ **	AntiV (A ***, seq ****)^ID^ **	AntiC (A ***, seq ****)^ID^ **
^I ^Legumin-like protein (1^T^) (356)^ID 1140^	(0.0084, 2xYG, YGG)^ID 1140^	-	(0.0028, YVL)^ID 1140^	-	-	(0.0028, VVV)^ID 1140^
^I ^Profilin-1 (1^T^) (131)^ID 1797^ Protein (1^T^) ***** Epidermal retin acid binding protein (EBP) (55)^ID 1200^	(0.0076, GLF)^ID 1797^	(0.0076, GFL)^ID 1797^	-	-	-	-
^II ^Cocoa storage protein (1^T^) (407)^ID 1114^	(0.0049, 2xYG)^ID 1114^	-	-	-	-	-
^III ^11S globulin (beta-legumin-like chain) (1^T^) (440)^ID 1143^	-	(0.0023, EAE)^ID 1143^	(0.0023, IAK)^ID 1143^	-	-	-
^IV ^11S globulin beta subunit (1^T^) (480)^ID 1161^	(0.0021, YG)^ID 1161^	(0.0021, EAE)^ID 1161^	-	-	-	-
^V ^11S globulin seed storage protein G3 (1^T^) (493,pr)^ID 1163^	-	(0.0020, LLY)^ID 1163^	-	-	-	(0.0041, 2xVVV)^ID 1163^
^VI ^Legumins (beta-globulins) (2^T^) (494)^ID 1141^, (488,pr)^ID 1164^	-	(0.0020, LLY)^ID 1164^	-	-	-	-
^VII ^Cruciferin, 11S globulin (1^T^) (486)^ID 1235^	-	-	-	-	-	-
^VIII ^Monellins, chain B (II), chain A (I) (2^T^) Proteins (2^T^) ***** (50)^ID 1169^, (45)^ID 1170^	-	-	-	-	-	-

ImmD—immunomodulatory, ImmS—immunostymulatory, AntiB—antibacterial, AntiF—antifungal, AntiV—antiviral, AntiC—anticarcinogenic activities, ^T^—the number of total proteins including all protein fragments available from the BIOPEP-UWM database (accessed on August/September 2022 and August/September 2023. The data were updated as of 1 October 2023), pr—precursor, f—fragment of protein, * NAAR—the number of amino acid residues found in the analyzed protein or protein fragment, ** ID—identification number in the BIOPEP-UWM database, *** A—the A parameter (the frequency of bioactive fragment occurrence in a protein sequence, defined in the “Materials and Methods” section), **** seq—sequence of biopeptides, amino acid sequences presented in the form of a single-letter amino acid code: A—alanine, E—glutamic acid, G—glycine, I—isoleucine, L—leucine, K—lysine, F—phenylalanine, Y—tyrosine, V—valine, ***** Protein(s)—a protein or a protein fragment not meeting the molecular criterion, i.e., possessing at least 100 amino acid residues.
